# Room-temperature quantum optomechanics using an ultralow noise cavity

**DOI:** 10.1038/s41586-023-06997-3

**Published:** 2024-02-14

**Authors:** Guanhao Huang, Alberto Beccari, Nils J. Engelsen, Tobias J. Kippenberg

**Affiliations:** 1 Institute of Physics, Swiss Federal Institute of Technology Lausanne (EPFL), Lausanne, Switzerland; 2 Center for Quantum Science and Engineering, Swiss Federal Institute of Technology Lausanne (EPFL), Lausanne, Switzerland; 3 Department of Microtechnology and Nanoscience (MC2), Chalmers University of Technology, Göteborg, Sweden

**Keywords:** Optomechanics, Quantum physics, Quantum metrology, Applied physics

## Abstract

At room temperature, mechanical motion driven by the quantum backaction of light has been observed only in pioneering experiments in which an optical restoring force controls the oscillator stiffness^1,2^. For solid-state mechanical resonators in which oscillations are controlled by the material rigidity, the observation of these effects has been hindered by low mechanical quality factors, optical cavity frequency fluctuations^3^, thermal intermodulation noise^4,5^ and photothermal instabilities. Here we overcome these challenges with a phononic-engineered membrane-in-the-middle system. By using phononic-crystal-patterned cavity mirrors, we reduce the cavity frequency noise by more than 700-fold. In this ultralow noise cavity, we insert a membrane resonator with high thermal conductance and a quality factor (*Q*) of 180 million, engineered using recently developed soft-clamping techniques^6,7^. These advances enable the operation of the system within a factor of 2.5 of the Heisenberg limit for displacement sensing^8^, leading to the squeezing of the probe laser by 1.09(1) dB below the vacuum fluctuations. Moreover, the long thermal decoherence time of the membrane oscillator (30 vibrational periods) enables us to prepare conditional displaced thermal states of motion with an occupation of 0.97(2) phonons using a multimode Kalman filter. Our work extends the quantum control of solid-state macroscopic oscillators to room temperature.

## Abstract (web summary)

A room-temperature demonstration of optomechanical squeezing of light and measurement of mechanical motion approaching the Heisenberg limit using a phononic-engineered membrane-in-the-middle cavity with ultralow noise.

## Main

The fragile nature of quantum systems renders them susceptible to the influence of the thermal environment^9^. This presents a substantial challenge for quantum science and technology, which is especially hard to overcome for solid-state systems. Nevertheless, over the past decade, quantum control has been extended to solid-state mechanical resonators, both with radiation pressure optomechanical coupling^8^ and piezoelectric coupling with superconducting qubits^10,11^. Cavity optomechanics, in which the mechanical oscillator is dispersively coupled to an optical cavity, has enabled numerous advances, including ground state cooling^12,13^, optomechanical squeezing of light^14–19^ and entanglement of separate mechanical oscillators^20–22^. Yet, all these advances necessitate cryogenic cooling to reduce thermal fluctuations. Room-temperature operation is beneficial to the accessibility and widespread adoption of technology, as witnessed in other branches of physical science^23–25^. Developing room-temperature quantum optomechanical systems would imply a drastic reduction in experimental complexity by removing the limitations imposed by cryocoolers such as poor thermalization, excess acoustic noise and limited optical access. Room-temperature operation could stimulate applications such as coupling to atomic systems^26^, force microscopy^27^ and variational displacement measurements^18^.

To enter the quantum regime of optomechanics, the product between the total force noise ${\bar{S}}_{{\rm{FF}}}^{{\rm{tot}}}$ (including environment thermal force ${\bar{S}}_{{\rm{FF}}}^{{\rm{th}}}$ as well as measurement-induced backaction ${\bar{S}}_{{\rm{FF}}}^{{\rm{ba}}}$) and the displacement measurement imprecision ${\bar{S}}_{xx}^{{\rm{imp}}}$ must approach the limit $\sqrt{{\bar{S}}_{xx}^{{\rm{imp}}}{\bar{S}}_{{\rm{FF}}}^{{\rm{tot}}}}\ge \hbar /2$ set by the Heisenberg uncertainty principle^8^. A necessary condition imposed by this limit is that the quantum backaction (QBA) rate from the light field ${\varGamma }_{{\rm{qba}}}={x}_{{\rm{zpf}}}^{2}{\bar{S}}_{{\rm{FF}}}^{{\rm{qba}}}/{\hbar }^{2}$ (where *x*_zpf_ is the zero-point displacement fluctuation amplitude of the oscillator) must exceed the thermal decoherence rate ${\varGamma }_{{\rm{th}}}={x}_{{\rm{zpf}}}^{2}{\bar{S}}_{{\rm{FF}}}^{{\rm{th}}}/{\hbar }^{2}$ of the mechanical oscillator, which is determined by the bath temperature *T* and by the quality factor *Q* as *Γ*_th_ = *k*_B_*T*/(*ħQ*). This condition is characterized by the quantum cooperativity ${{\mathcal{C}}}_{{\rm{q}}}={\varGamma }_{{\rm{qba}}}/{\varGamma }_{{\rm{th}}}$ of the system.

Over the past decade, several approaches have been made to reach the ultralow mechanical dissipation required to enter the quantum regime at room temperature, including levitated nanoparticles^28^ and micromechanical objects whose rigidity is controlled by an optical field^1,29^. These methods enhance the mechanical *Q* by optical trapping and resulted in the recent observations of quantum backaction^1^, optomechanical squeezing of light^17,30^ and ground state cooling^2^. Yet, room-temperature quantum optomechanical phenomena have not been accessible with engineered solid-state mechanical resonators^3,4,31^ because of thermal intermodulation noise^4^, vibrations of the cavity mirror substrates^3^ and optical heating-induced instability^32^. These thermal effects result in excess imprecision and backaction noise, preventing their product from reaching the Heisenberg limit.

Here we overcome these challenges and demonstrate optomechanical squeezing of light at room temperature using a phononic-engineered membrane-in-the-middle (MIM) system. Furthermore, through efficient measurement of mechanical motion, we prepare displaced thermal states with single-phonon occupation. This implies that the measurement efficiency is sufficient to implement measurement-based quantum state preparation protocols, for example, feedback cooling to the ground state^33^.

## Ultralow noise optomechanical cavity

**Fig. 1:**
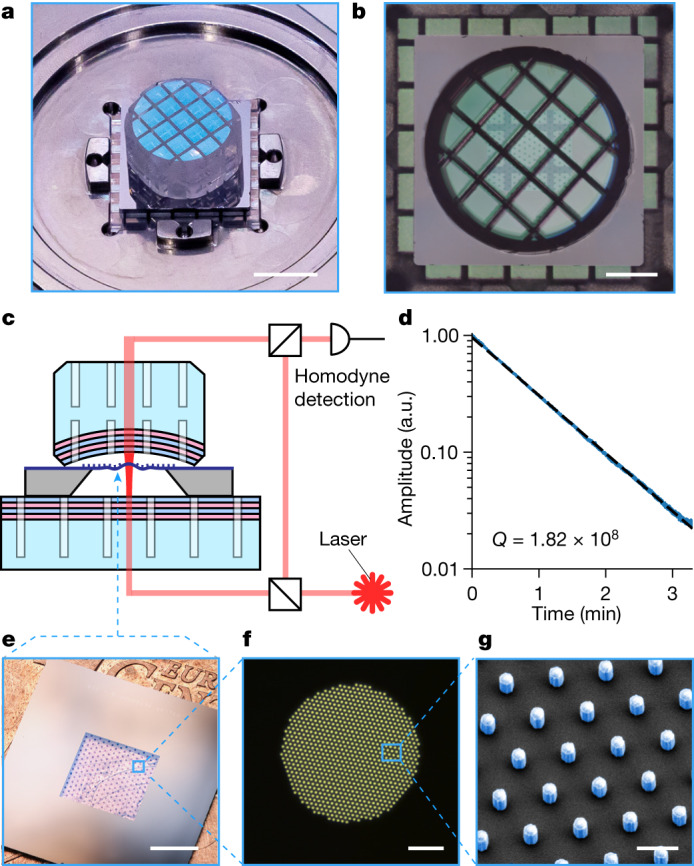
Ultralow noise phononic-engineered membrane cavity. **a**, Photograph of the membrane-in-the-middle assembly. **b**, Optical microscope image of the MIM assembly from the top, showing the overlapping square unit cells of the top and bottom phononic crystal mirrors and the density-modulated membrane. **c**, Setup schematic. **d**, Mechanical ringdown measurement of the quality factor of the soft-clamped mode of the pillar membrane. The ringdown was acquired with the membrane installed in the MIM cavity. **e**–**g**, Overview images and details of a pillar membrane sample at different length scales. Scale bars, 3 mm (**a**); 2 mm (**b**,**e**); 25 μm (**f**); and 1 μm (**g**).

To achieve Heisenberg-limited operation, we adopt a modular approach and use the MIM architecture^34^ using an optical Fabry–Pérot cavity (Fig. 1a–c). The high-finesse cavity (${\mathcal{F}}\approx 1{0}^{4}$) enables operation at high *C*_q_ while keeping the optical probe power below 1 mW, at which the probe laser is quantum-limited in phase and amplitude noise. Heisenberg-limited operation further requires low displacement measurement imprecision, that is, ${\bar{S}}_{xx}^{{\rm{imp}}} < {x}_{{\rm{zpf}}}^{2}/{\varGamma }_{{\rm{th}}}$. This is particularly challenging at room temperature, as the required imprecision scales inversely with temperature. For our device, we estimate the bound to be 10^−35^ m^2^ Hz^−1^. The cavity frequency noise is thus required to be extremely small and should satisfy ${\bar{S}}_{\nu \nu }(\,f) < {({g}_{0}/2{\rm{\pi }})}^{2}/{\varGamma }_{{\rm{th}}}$ (where *g*_0_ is the vacuum optomechanical coupling rate) to enable ground state cooling^35^ and significant optomechanical squeezing. This bound is well below the typical thermal fluctuations of the cavity mirrors, even with state-of-the-art mechanical resonators. Suppression of the driven response of the mirror has been achieved with phononic shielding^3^, but a significant reduction of thermomechanical cavity mirror noise has remained elusive.

**Fig. 2:**
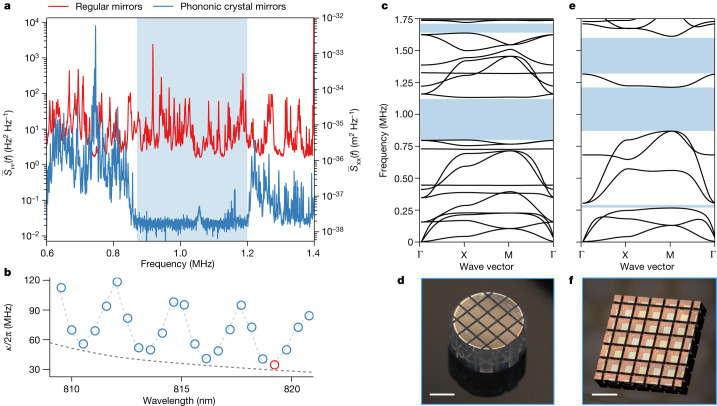
Suppression of cavity frequency noise in the phononic bandgap. **a**, Cavity frequency noise comparison between regular mirror assembly and phononic crystal mirror assembly, showing a 700-fold total noise reduction in the blue-shaded region. The noise is measured with a silicon spacer chip in place of the membrane chip inside the cavity. The vertical axis is calibrated in cavity frequency noise units (left) and in equivalent mirror mechanical displacement units (right). **b**, MIM cavity optical linewidth as a function of wavelength. The blue circles indicate measured optical cavity linewidths. The red circle indicates the optical mode used for experiments. The modulation of the cavity linewidth is because of the presence of the membrane in the cavity. The dashed grey line indicates the ideal empty cavity linewidth based on the measured mirror transmission after the deposition of the high-reflectivity coating but before the definition of the PNC. **c**,**d**, Band diagram and photograph of the top phononic crystal mirror. **e**,**f**, Band diagram and photograph of the bottom phononic crystal mirror. Scale bar, 3 mm (**d**,**f**).

We overcome this challenge by engineering the vibrational spectra of the cavity mirrors with phononic crystal structures (PNC) (Fig. 2d,f). A precision circular saw is used to pattern the phononic structure on glass mirror substrates endowed with high-reflectivity dielectric coatings. The phononic unit cell dimensions (Supplementary Information) are chosen such that mirror motion in the frequency band of 0.87–1.2 MHz is prohibited (Fig. 2c,e). The thermomechanical noise density ${\bar{S}}_{\nu \nu }(\,f)$ in this band is reduced by a factor of more than 700 as shown in Fig. 2a (measurement limited by laser noise). This noise reduction greatly relaxes the requirements to observe quantum optomechanical effects at room temperature. The phononic crystal patterning did not result in significant excess optical losses, as the membrane-loaded cavity linewidths are consistent with the ideal unpatterned cavity linewidths (Fig. 2b), thereby maintaining high cavity out-coupling efficiency *η*_cav_. We use the optical mode at 819 nm for the experiment, which has *η*_cav_ > 80% with an optical linewidth of *κ*/2π = 34.2 MHz.

A suitable ultracoherent membrane resonator is vital for the operation of the MIM system at room temperature. To this end, phononic density-modulated membranes are promising^7^ as they maintain higher material stress and thereby show enhanced dissipation dilution compared with stress-modulated, perforated membranes^6^. Furthermore, unperforated membranes benefit from increased heat dissipation, diminishing thermal effects due to optical absorption. However, after reproducing the design as in ref. ^7^, we found that the membrane had high optical absorption, which led to strong cavity bistability^36^ and mechanical instability^32^. We, therefore, developed a fabrication process that minimizes optical absorption, such that the photothermal effects are absent for the optical mode and optical powers used in this experiment.

The mechanical resonator (Fig. 1e–g) consists of an Si_3_N_4_ membrane patterned with aSi-HfO_2_ nanopillars (700-nm diameter) much smaller than the acoustic wavelength, implementing phononic density modulation^7^. The periodic density modulation creates a mechanical bandgap that spectrally isolates a 7-ng high-*Q* soft-clamped defect mode with a mechanical frequency of *Ω*_m_/2π = 1.16 MHz and a damping rate of *Γ*_m_/2π = 6.41 mHz, corresponding to a room-temperature thermal occupancy of ${\overline{n}}_{{\rm{th}}}=5.3\times 1{0}^{6}$ and zero-point fluctuations of *x*_zpf_ = 1.0 fm. A ringdown measurement of mechanical *Q* = 1.8 × 10^8^ is shown in Fig. 1d. More details on the device fabrication are given in the Methods. By clamping the density-modulated membrane chip in between the phononic crystal mirrors, we construct an MIM system with *g*_0_/2π = 160 Hz and cavity frequency noise satisfying the ${\bar{S}}_{\nu \nu }(\,f) < 0.11\,{{\rm{Hz}}}^{2}\,{{\rm{Hz}}}^{-1}$ requirement, which enables high quantum cooperativity operation with quantum-noise-limited measurement imprecision and backaction.

## Optomechanical squeezing of light

To demonstrate that the system operates in the quantum regime at room temperature, we generate optomechanical squeezing—the quantum signature most robust against calibration errors. In the textbook description of cavity optomechanics, the mechanical motion is driven by the vacuum fluctuations of the laser amplitude and transduced by the linear response of the cavity into phase fluctuations of the light field. The induced phase–amplitude correlation of the light field manifests as a noise reduction below the shot noise level (squeezing). However, the nonlinear transduction response of the cavity produces mixing products at the sum and difference frequencies of the mechanical modes, giving rise to excess nonlinear noise that does not naturally fit in the linear framework of optical quadratures. Owing to the high number of modes and their large Brownian motions at room temperature, the mixing products manifest as broadband noise, termed thermal intermodulation noise (TIN)^4^. TIN results in intracavity photon number fluctuations that have significant power even at frequencies within the mechanical bandgap, thereby degrading the measurement signal-to-noise ratio, and inducing additional mechanical decoherence^5^.

**Fig. 3:**
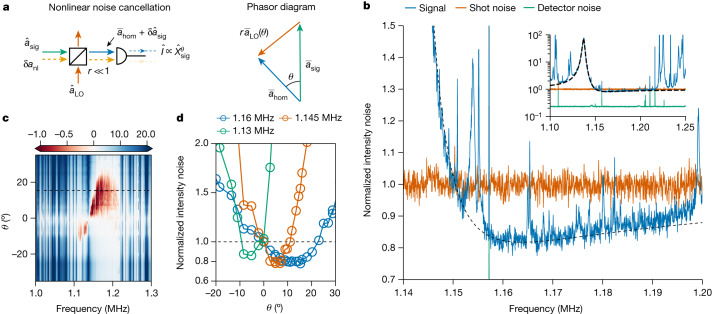
Optomechanical generation of squeezed light. **a**, Homodyne detection scheme that cancels nonlinear mixing noise *δa*_nl_ from the records of cavity transmission. For each quadrature angle *θ*, a specific local oscillator amplitude is needed to cancel the nonlinear mixing noise. **b**, Detected power spectral density (PSD) at the quadrature angle *θ* = 15.3° (black dashed line in **c**) at frequencies above the mechanical resonance, compared with the measured shot noise and detector noise. Inset, the same PSD is shown on broader frequency and power ranges. **c**, Collection of homodyne photocurrent spectra (in dB scale) of the cavity output field, normalized to the shot noise level, measured at different quadrature angles. The magnitude and bandwidth of squeezing depend on the homodyne quadrature angle. **d**, Averaged homodyne PSD within three different frequency bands (5 kHz bandwidth) at different quadrature angles, showing a maximum squeezing depth of 22.2(3)% (1.09(1) dB).

To eliminate TIN intracavity photon number fluctuations, we pump the cavity with the laser detuned by $2\varDelta /\kappa =-1/\sqrt{3}$ (magic detuning; where *Δ* is the laser detuning from cavity resonance), in which the quadratic term of the cavity response vanishes. This operation has the additional effect of cooling the defect mode to an occupancy ${\bar{n}}_{{\rm{eff}}}\approx 20$ phonons, by dynamical backaction cooling^8^ (a lower effective phonon occupancy of ${\bar{n}}_{{\rm{eff}}}\approx 5.7$ was achieved with a narrower-linewidth cavity mode at 862 nm; Supplementary Information). To eliminate TIN in the optical detection, we deploy a specialized homodyne detection scheme using only one detector (Fig. 3a). Instead of balanced homodyne detection, a single detector offers the required photodetection nonlinearity^4^ to eliminate TIN at arbitrary optical quadrature angles, by carefully selecting the local oscillator power for each quadrature angle. A detailed description is provided in the Methods.

We measure the noise of the cavity output field at optical quadrature angles ranging from −33° to 33° (Fig. 3c), where the 0° quadrature is defined as the one with no mechanical displacement information. Depending on the quadrature angle, we observed optical squeezing (up to 50 kHz bandwidth) on either side of the defect mode, the extent of which is limited by the membrane modes at the edge of the bandgap (see Fig. 3b for a representative spectrum). For the three frequency bands that are devoid of parasitic modes, we compute the average intensity noise over a bandwidth of 5 kHz at different quadrature angles (Fig. 3d) and observe a maximum squeezing of 22.2(3)% (1.09(1) dB) below the shot noise level. More details on the shot noise calibration are described in the Supplementary Information.

## Conditional quantum state preparation

The observation of optomechanical squeezing demonstrates that we can conduct quantum measurements with high efficiency. With quantum-limited detection, the maximum squeezing equals the measurement efficiency *η*_meas_ = *Γ*_meas_/(*Γ*_th_ + *Γ*_qba_) of the system, with ${\varGamma }_{{\rm{meas}}}={x}_{{\rm{zpf}}}^{2}/\left(4{\bar{S}}_{xx}^{{\rm{imp}}}\right)$ being the measurement rate^37^, which also quantifies how far the measurement is from the Heisenberg uncertainty limit: $\sqrt{{\bar{S}}_{xx}^{{\rm{imp}}}{\bar{S}}_{{\rm{FF}}}^{{\rm{tot}}}}=\hbar /\left(2\sqrt{{\eta }_{{\rm{meas}}}}\right)$. Measurement efficiency is likewise crucial for measurement-based quantum control of mechanical motion^13^ and determines the purity of the prepared mechanical states. We prepare conditional mechanical states by measuring the mechanical resonator at a rate close to its decoherence rate, demonstrating that our system is in a parameter regime in which quantum control of mechanical motion is possible at room temperature.

We proceed by stabilizing the laser at the magic detuning and adjusting the single-detector homodyne to measure the mechanical motion at the quadrature angle *θ* ≈ −90°, maximizing the readout efficiency of mechanical motion. We digitize the measurement signal at a 14-MHz rate over 2 s for state preparation in post-processing. By fitting the measured noise spectrum with our model, we extract a total detection efficiency of *η*_d_ = 31% and ${{\mathcal{C}}}_{{\rm{q}}}=0.93$ (Supplementary Information). These parameters correspond to a measurement rate of *Γ*_meas_ = *η*_d_*Γ*_qba_ = 2π × 11 kHz, approaching the thermal decoherence rate of *Γ*_th_ = 2π × 34 kHz and resulting in a measurement efficiency of *η*_meas_ = 16%. This efficiency corresponds to an imprecision-force noise product 2.5 times the Heisenberg uncertainty limit. The degradation of the measurement efficiency compared with the condition for maximum squeezing mainly comes from the lower homodyne efficiency at a quadrature angle of *θ* ≈ −90°.

**Fig. 4:**
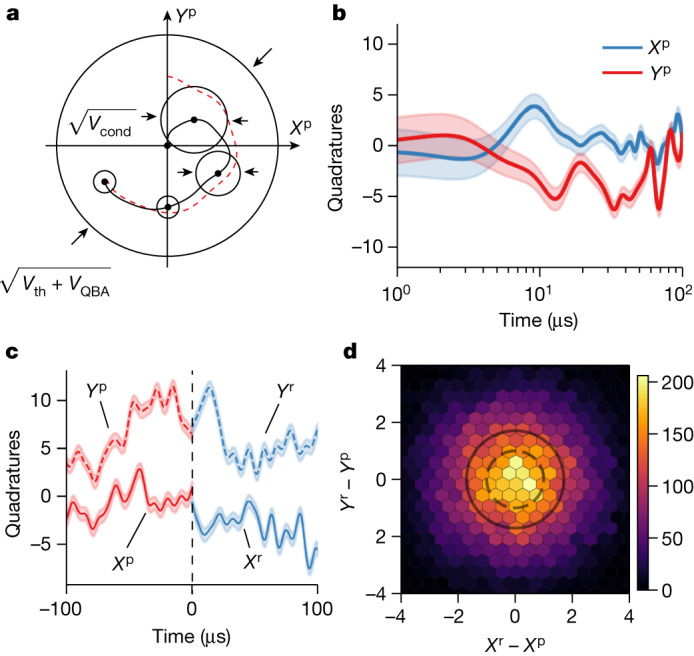
Conditional state preparation and verification. **a**, Schematic of state preparation by continuous displacement measurement. The mechanical mode is initially in a thermal state assuming no previous information. The state is continuously estimated and purified over time given the measurement record (red dashed line). **b**, Exemplary quantum trajectory of the mechanical quadratures **r**^**p**^ = (*X*^p^, *Y*^p^). The shaded width corresponds to 1*σ* of uncertainty in the estimated quadratures. **c**, Using state retrodiction **r**^**r**^ = (*X*^r^, *Y*^r^) (blue), the differences with the prediction result **r**^**p**^ (red) at *t* = 0 are used to reconstruct the covariance matrix of the multimode system. **d**, Phase-space density heat map of the collective mode of interest, with the solid circle marking the statistical standard deviation, and the dashed circle indicating the standard deviation of a pure coherent state as a reference.

As is shown in Fig. 4a, the mechanical mode is initially in a thermal state with phase-space variance determined by both thermal decoherence and QBA decoherence. Based on the continuous measurement record, optimal state estimation predicts both the most probable values of the mechanical quadratures **r**^**p**^ = (*X*^p^, *Y*^p^) and the corresponding uncertainties in time. However, as there are parasitic modes near the mode of interest, single-mode state estimation^2,38^ underestimates the conditional occupancy. We, therefore, conduct multimode Kalman filtering based on the quantum master equations of the system that include the nine nearest modes (details of calibration are provided in the Supplementary Information). Using this method, we can isolate the mechanical motion of the defect mode (Fig. 4b) and mitigate spectral contamination between different modes.

To estimate the quadrature variances *V*_*X*,*Y*_, we follow a retrodiction procedure^39,40^ (Methods), using the measurement records in the ‘future’ relative to the time of state conditioning as a separate result **r**^**r**^ (Fig. 4c). The quadrature variance between the prediction and retrodiction results is exactly ⟨⟨∥**r**^**r**^ − **r**^**p**^‖^2^⟩⟩ = 4*V*_*X*,*Y*_, where ⟨⟨⋯⟩⟩ is the statistical average over the dataset. We retrieved a thermal occupation $${\overline{n}}_{{\rm{cond}}}\,=$$$${V}_{X,Y}-1/2=1.43(3)$$ of the prepared state, with only a 3% deviation from the theoretical value. This multimode estimation result shows a 63% increased thermal occupancy compared with the idealized single-mode estimation. The degradation stems from the fact that mechanical modes cannot be fully distinguished from each other because of finite spectral overlap. This results in strong cross-correlations between mechanical modes, because of which collective modes with enhanced measurement rates can be defined^41^. From the reconstructed multimode covariance matrix *C*, we can define a set of uncorrelated collective modes that diagonalizes *C* through a symplectic transformation (for details see the Methods). We conduct a similar prediction–retrodiction procedure with the collective modes, and plot the quadrature differences **r**^**r**^ − **r**^**p**^ in Fig. 4d. We find a modified defect mode thermal occupancy of $${\overline{n}}_{{\rm{cond,col}}}=0.97(2)$$ in the new collective mode bases, with a corresponding state purity of 34.0(5)%.

## Discussion

Using an ultralow noise cavity in conjunction with a PNC density-modulated membrane, we have been able to operate in the quantum regime of cavity optomechanics at room temperature. With a single-detector homodyne scheme countering TIN, we demonstrate optomechanical squeezing and conditional state preparation of displaced thermal states with single-phonon occupation, which is a prerequisite for real-time quantum control of this macroscopic mechanical resonator.

With a reasonable improvement of the mechanical quality factor and a wider mechanical bandgap^7^, together with a real-time digital feedback using the studied optimal Kalman filters, we expect measurement-based feedback cooling to the ground state to be feasible with continuous^2,13^ or gated^20^ feedback. Non-Gaussian states at room temperature can also be prepared using nonlinear measurements, for example, photon counting^42^, which is inherently compatible with our nonlinear noise cancellation scheme.

In the long term, the ability to observe optomechanical squeezing at room temperature is advantageous for hybrid quantum systems^43^, with, for example, atomic ensembles^26,44^ and solid-state spins^45,46^, by obviating the need to operate experiments inside cryostats that have limited optical access and low thermal budget. Quantum control of macroscopic systems at room temperature may establish paths for practical applications in real-world scenarios^47,48^.

## Methods

### Fabrication of density-modulated membranes

We use the soft clamping^6,49–53^ technique to realize ultrahigh mechanical quality factors. Our membrane design is inspired by those pioneered in ref. ^7^, but we use a different material for the nanopillars and a different fabrication process (see Supplementary Information for more details). We fabricated density-modulated PNC membranes by patterning amorphous silicon (aSi) nanopillars on a high aspect ratio Si_3_N_4_ membrane. In our PNC membranes, we fabricated pillars with diameters *d*_pil_ = 300–800 nm, thickness of about 600 nm and nearest-neighbour distances *a*_pil_ = 1.0–2.0 μm. Amorphous silicon is grown with plasma-enhanced chemical vapour deposition (PECVD) at a temperature of 300 °C. Electron-beam lithography (FOx16 electron-beam resist) and dry etching (using a plasma of SF_6_ and C_4_F_8_) are used to pattern pillar arrays in aSi. Dry etching is stopped on a 6-nm layer of HfO_2_ (hafnium oxide) grown with atomic layer deposition (ALD) directly on top of Si_3_N_4_. HfO_2_ is used as an etch-stop layer because it is quite resistant to hydrofluoric acid (HF) etching, and the undercut created at the pillar base in the following process steps is limited. Undercut minimization is important to control the added dissipation induced by pillar motion (Supplementary Information). We remove the FOx mask and the residual etch-stop layer by dipping the wafer in HF 1% for about 3.5 min.

After patterning the pillars, we encapsulate them in a PECVD Si_*x*_N_*y*_ layer to protect them during the silicon deep etching step. We first grow a thin (about 20 nm), protective layer of Al_2_O_3_ with ALD, to shield the membrane layer from plasma bombardment during PECVD. Then, approximately 125 nm of Si_*x*_N_*y*_ is grown at 300 °C, with 40 W of radio-frequency power exciting the plasma during deposition. This Si_*x*_N_*y*_ layer has been characterized to have a tensile stress of around +300 MPa at room temperature. The layer perfectly seals the nanopillars during immersion in hot KOH, without significant consumption.

After patterning the pillars on the wafer frontside film, a thick (about 3 μm) layer of positive tone photoresist is spun on top for protection during the backside lithography process, which we perform with an MLA150 laser writer (Heidelberg Instruments). Optical lithography is followed by Si_3_N_4_ dry etching with a plasma of CHF_3_ and SF_6_. After the resist mask and protection layer removal with *N*-methyl-2-pyrrolidone (NMP) and O_2_ plasma, we deep-etch with KOH from the membrane windows while keeping the frontside protected, by installing the wafer in a watertight PEEK holder in which only the backside is exposed^6^. KOH 40% at 70 °C is used, and the etch is interrupted when about 30–40 μm of silicon remains. The wafer is then rinsed and cleaned with hot HCl of the residues formed during KOH etching. Then, the wafer is separated into individual dies with a dicing saw, and the process continues chipwise. Chips are again cleaned with NMP and O_2_ plasma, and the deep-etch is concluded with a second immersion in KOH 40% at a lower temperature of 55 °C, followed by cleaning in HCl. From the end of the KOH etching step, the composite membranes are suspended, and great care must be taken while displacing and immersing the samples in liquid. We dry the samples by moving them to an ultrapure isopropyl alcohol bath after water rinsing. Isopropyl alcohol has a high vapour pressure, and quickly evaporates from the chip interfaces, with few residues left behind.

Finally, the PECVD nitride and Al_2_O_3_ layers can be removed selectively with wet etching in buffered HF. The chips are loaded in a Teflon carrier in which they are vertically mounted and immersed for about 3 min 20 s in BHF 7:1. It is crucial not to etch more than necessary to fully remove the encapsulation films: membranes become extremely fragile and the survival yield drops sharply when their thickness is reduced below around 15 nm. The membranes are then carefully rinsed, transferred in an ethanol bath and dried in a critical point dryer, in which the liquids can be evacuated gently and with little contamination.

### Fabrication and simulation of phononic-crystal-patterned mirrors

The top and bottom mirror substrates are, respectively, fused silica and borosilicate glass, with a high-reflection coating sputtered on one face and an anti-reflection layer coating the other face. No layer for the protection of the optical coating is applied before machining. We use a dicing saw for glass machining to pattern a regular array of lines into the mirror substrates. The blade is continuously cooled by a pressurized water jet during the patterning process. The maximum cut depth allowed for our blade is 2.5 mm, and we constrain the designed PNC accordingly. We cut the flat bottom mirror from only one side (its thickness is only 1 mm), and the top mirror is patterned symmetrically with parallel cuts from both sides, as it is 4 mm thick. The relatively deep cuts in the top mirror need to be patterned over several passes, with gradually increasing depths. After patterning one mirror side, the piece is flipped and the other side is patterned after aligning to the first cuts, visible through the glass substrate. The lines are arranged in a square lattice for simplicity, although more complex patterns can be machined with the dicing saw. After the dicing process, the mirrors are subject to ultrasonic cleaning, while immersing first in acetone and then in isopropanol.

We simulate the band diagrams of the unit cells of both the top and the bottom mirrors in COMSOL Multiphysics with the Structural Mechanics module. We optimized the lattice constant and cut depths to maximize the bandgap width, while centring the bandgap around 1 MHz and making sure that the remaining glass thickness is sufficient to maintain a reasonable level of structural stiffness. Details of the PNC dimensions are shown in the Supplementary Information. Owing to the finite size of the mirrors, we expect to observe edge modes within the mechanical bandgap frequency range. The thermal vibrations of these modes penetrate into the PNC structure with exponentially decaying amplitudes. To account for their noise contributions, we simulated the frequency noise spectrum of the MIM assembly (details shown in the Supplementary Information). The eigenfrequency solution confirmed the existence of edge modes with frequencies within the mechanical bandgap, but did not predict any significant contribution to the cavity frequency noise: the PNC is sufficiently large to reduce their amplitude at the cavity mode position.

After patterning the PNC structures on the mirrors, we assembled a cavity with a spacer chip in place of a membrane and observed that the TE_00_ linewidth with the diced mirrors is identical to that of the original cavity. This indicates that our fabrication process does not cause measurable excess roughness or damage to the mirror surfaces. By contrast, when the assembly was clamped too tightly, excess cavity loss occurred because of significant deformation of the PNC mirrors, with a reduced stiffness. We mitigate this detrimental effect in the experiment by gently clamping the MIM cavity, with a spring compression sufficient to guarantee the structural stability of the assembly. We also ensure that the cavity mode is well-centred on the bottom mirror, to reduce the thermal noise contribution of the upper band-edge modes. For the MIM experiment discussed in the main text, we did not observe any mirror modes within the mechanical bandgap of the membrane chip. We can distinguish membrane modes from mirror modes by exploiting the fact that the coupling rates of membrane modes vary between different cavity resonances, whereas this is not the case for mirror modes.

### Nonlinear noise cancellation scheme

At room temperature, the large thermal noise of the cavity, combined with the nonlinear cavity transduction response, results in a nonlinear mixing noise (TIN). This noise could lead to excess intracavity photon fluctuations and also to excess noise in optical detection. In the following, we discuss the strategy to cancel these effects in the fast-cavity limit (*ω* ≪ *κ*). Theoretical derivations and a discussion of the effect of a finite *ω*/*κ* ratio can be found in the Supplementary Information.

In the experiment, we pump the cavity at the magic detuning, $2\overline{\varDelta }/\kappa =-1/\sqrt{3}$, in which the nonlinear photon number noise is cancelled, to prevent excess oscillator heating due to nonlinear classical radiation pressure noise. To show the quantum correlations leading to optomechanical squeezing and conduct measurement-based state preparation, we need to perform measurements at arbitrary optical quadrature angles. Balanced homodyne detection provides the possibility of tuning the optical quadrature, but it does not offer enough degrees of freedom to cancel the nonlinear noise in detection. However, if the local oscillator is injected from a highly asymmetric beam splitter with a very small reflectivity (*r* ≪ 1) and the combined field is detected on a single photodiode, the photodetection nonlinearity is maintained and offers enough degrees of freedom to cancel the nonlinear noise in detection^4^ (for a derivation, see Supplementary Information). Specifically, simultaneous tuning of local oscillator amplitude and phase enables nonlinear mixing noise cancellation at arbitrary quadrature angles. In the fast-cavity limit, the cancellation condition is

$$\begin{array}{l}\left|\frac{{\overline{a}}_{{\rm{sig}}}}{{\overline{a}}_{\hom }}\right|=2{\rm{Re}}\,\left[\frac{{{\rm{e}}}^{-{\rm{i}}\theta }}{{(-{\rm{i}}\overline{\varDelta }+\kappa /2)}^{2}}\right]\,\left[{\overline{\varDelta }}^{2}+{\left(\frac{\kappa }{2}\right)}^{2}\right]\\ \,=2\cos [\theta -2\arg ({\chi }_{{\rm{cav}}}(0))],\end{array}$$

where ${\overline{a}}_{\hom }\approx {\overline{a}}_{{\rm{sig}}}+r{\overline{a}}_{{\rm{LO}}}$ is the coherent combination of the signal field ${\overline{a}}_{{\rm{sig}}}$ and the local oscillator field ${\overline{a}}_{{\rm{LO}}}$ (defined as the field before the beam splitter), *θ* = *θ*_hom_ − *θ*_sig_ is the quadrature rotation angle and ${\chi }_{{\rm{cav}}}(0)={\left(\kappa /2-i\overline{\varDelta }\right)}^{-1}$ is the cavity d.c. optical susceptibility.

In the experiment, to detect a certain quadrature angle while cancelling nonlinear noise, we lock the homodyne power at the corresponding combined field level ${I}_{\hom }=| {\overline{a}}_{\hom }{| }^{2}$. We then continuously vary the local oscillator power using a tunable neutral density filter until the noise in the mechanical bandgap is perfectly cancelled. The level of mixing noise is very sensitive to the local oscillator power, and therefore the cancellation point can serve as a good indicator of the measured quadrature angle *θ*. Knowing the field amplitudes $| {\overline{a}}_{\hom }| ,| {\overline{a}}_{{\rm{sig}}}|$ and that $\overline{\varDelta }=-\kappa /(2\sqrt{3})$, we can reconstruct the measured quadrature angle as the one satisfying the cancellation condition.

A detailed characterization of the nonlinear mixing noise and an analysis of single-detector homodyne efficiency can be found in the Supplementary Information.

### Multimode Kalman filter

The continuous position measurement of an oscillator at frequency *Ω*_m_ can be viewed as a form of heterodyne measurement of two orthogonal mechanical quadratures of motion $\widehat{X}$ and $\widehat{Y}$ that rotate with frequency *Ω*_m_. IQ demodulation can then be carried out at the mechanical frequency *Ω*_m_. This results in two independent measurement channels of two orthogonal mechanical quadratures with independent measurement noise.

We work in a parameter regime in which the measurement rate is significantly smaller than the frequency of the mechanical mode, such that we can perform IQ demodulation of the mechanical motion at *Ω*_m_ to obtain the slowly varying $\widehat{X},\widehat{Y}$ quadratures. Their evolution is described by decoupled quantum master equations^33^. In this parameter regime, only thermal coherent states are prepared through the measurement process. These states are essentially thermal states displaced from the origin of the phase space and belong to the larger group of Gaussian states.

We operate in the fast-cavity limit *Ω*_m_ ≪ *κ*, so the cavity dynamics are simplified in our modelling. After IQ demodulation, the normalized photocurrent signal is described by

1 $${\bf{i}}(t){\rm{d}}t={\rm{d}}{\bf{W}}(t)+\sum _{i}\sqrt{4{\varGamma }_{{\rm{meas}}}^{i}}\langle {\widehat{{\bf{r}}}}_{i}\rangle (t){\rm{d}}t$$

where the subscript *i* denotes different mechanical modes, $${\bf{i}}=\left[\begin{array}{c}{i}_{X}\\ {i}_{Y}\end{array}\right]$$, $${\widehat{{\bf{r}}}}_{i}=\left[\begin{array}{c}{\widehat{X}}_{i}\\ {\widehat{Y}}_{i}\end{array}\right]$$ and $${\rm{d}}{\bf{W}}=\left[\begin{array}{c}{\rm{d}}{W}_{X}\\ {\rm{d}}{W}_{Y}\end{array}\right]$$. The Wiener increment d*W*_*X*,*Y*_(*t*) = *ξ*(*t*)d*t* is defined in terms of an ideal unit Gaussian white noise process $\langle \xi (t)\xi ({t}^{{\prime} })\rangle =\delta (t-{t}^{{\prime} })$.

As the measurement is purely linear, the system remains in a Gaussian state^54^, and the dynamics are completely captured by the expectation values of the quadratures ⟨*X*_*i*_⟩, ⟨*Y*_*i*_⟩ and their covariance matrix *C*. We derive the time evolution of the quadrature expectation values as

2 $${\rm{d}}\langle {\widehat{{\bf{r}}}}_{i}\rangle ={A}_{i}\langle {\widehat{{\bf{r}}}}_{i}\rangle {\rm{d}}t+2{B}_{i}{\rm{d}}{\bf{W}}(t),$$

where

$${A}_{i}=\left[\begin{array}{cc}-{\varGamma }_{{\rm{m}}}^{i}\,/2 & {\varOmega }_{i}-{\varOmega }_{{\rm{m}}}\\ {\varOmega }_{{\rm{m}}}-{\varOmega }_{i} & -{\varGamma }_{{\rm{m}}}^{i}\,/2\end{array}\right]$$

and

$${B}_{i}=\left[\begin{array}{cc}{\sum }_{j}\sqrt{{\varGamma }_{{\rm{meas}}}^{j}}{C}_{{\widehat{X}}_{i}{\widehat{X}}_{j}} & {\sum }_{j}\sqrt{{\varGamma }_{{\rm{meas}}}^{j}}{C}_{{\widehat{X}}_{i}{\widehat{Y}}_{j}}\\ {\sum }_{j}\sqrt{{\varGamma }_{{\rm{meas}}}^{j}}{C}_{{\widehat{Y}}_{i}{\widehat{X}}_{j}} & {\sum }_{j}\sqrt{{\varGamma }_{{\rm{meas}}}^{j}}{C}_{{\widehat{Y}}_{i}{\widehat{Y}}_{j}}\end{array}\right].$$

The covariance matrix elements ${C}_{\widehat{M}\widehat{N}}=\langle \widehat{M}\widehat{N}+\widehat{N}\widehat{M}\rangle /2-\langle \widehat{M}\rangle \langle \widehat{N}\rangle$ evolve as

3 $$\begin{array}{l}{\dot{C}}_{{\widehat{M}}_{i}{\widehat{N}}_{j}}=-\frac{{\varGamma }_{{\rm{m}}}^{i}+{\varGamma }_{{\rm{m}}}^{j}}{2}{\dot{C}}_{{\widehat{M}}_{i}{\widehat{N}}_{j}}+{\delta }_{{\widehat{M}}_{i},{\widehat{N}}_{j}}{\varGamma }_{{\rm{th}}}^{i}+{\delta }_{M,N}\sqrt{{\varGamma }_{{\rm{qba}}}^{i}{\varGamma }_{{\rm{qba}}}^{j}}\\ \,\,+{(-1)}^{{\delta }_{M,Y}}({\varOmega }_{i}-{\varOmega }_{{\rm{m}}}){C}_{{\widehat{{\mathcal{M}}}}_{i}{\widehat{N}}_{j}}+{(-1)}^{{\delta }_{N,Y}}({\varOmega }_{j}-{\varOmega }_{{\rm{m}}}){C}_{{\widehat{M}}_{i}{\widehat{{\mathcal{N}}}}_{j}}\\ \,-4\left(\sum _{k}\sqrt{{\varGamma }_{{\rm{meas}}}^{k}}{C}_{{\widehat{M}}_{i}{\widehat{X}}_{k}}\right)\left(\sum _{l}\sqrt{{\varGamma }_{{\rm{meas}}}^{l}}{C}_{{\widehat{N}}_{j}{\widehat{X}}_{l}}\right)\\ \,-4\left(\sum _{k}\sqrt{{\varGamma }_{{\rm{meas}}}^{k}}{C}_{{\widehat{M}}_{i}{\widehat{Y}}_{k}}\right)\left(\sum _{l}\sqrt{{\varGamma }_{{\rm{meas}}}^{l}}{C}_{{\widehat{N}}_{j}{\widehat{Y}}_{l}}\right),\end{array}$$

where $\widehat{{\mathcal{M}}}$ and $\widehat{{\mathcal{N}}}$ are the canonical conjugate observables of $\widehat{M}$ and $\widehat{N}$.

Equations (1)–(3) form a closed set of update equations given the measurement record *i*(*t*), and enable quadrature estimations of an arbitrary number of modes and their correlations. The thermal occupancy ${\bar{n}}_{{\rm{c}}{\rm{o}}{\rm{n}}{\rm{d}},i}$ of a specific mechanical mode is determined by the quadrature phase-space variances ${V}_{{\widehat{X}}_{i}}={C}_{{\widehat{X}}_{i}{\widehat{X}}_{i}}$ and ${V}_{{\widehat{Y}}_{i}}={C}_{{\widehat{Y}}_{i}{\widehat{Y}}_{i}}$, which are both equal to ${\bar{n}}_{{\rm{c}}{\rm{o}}{\rm{n}}{\rm{d}},i}+1/2$.

We record the voltage output from the photodetector using an UHFLI lock-in amplifier (Zurich Instruments), digitizing the signal at a 14-MHz sampling rate for a total duration of 2 s, and we store the data digitally for post-processing. The noise power spectrum density of the digitized signal is compared with that simultaneously measured on a real-time spectrum analyser, to rule out signal-to-noise ratio degradation from the digitization noise. Details of an additional filtering step are discussed in the Supplementary Information. After filtering, only the 10 mechanical modes around the defect mode frequency *Ω*_m_ are kept for the multimode state estimation study.

To perform the multimode state estimation, we extract the required system parameters of the nearest 10 mechanical modes around *Ω*_m_ by fitting the measured spectral noise density. We demodulate the signal at *Ω*_m_ and feed the time-series signal **i**(*t*) to the discretized version of the update equation (2),

4 $$\Delta \langle {\widehat{{\bf{r}}}}_{i}\rangle ={A}_{i}^{{\prime} }\langle {\widehat{{\bf{r}}}}_{i}\rangle \Delta t+2{B}_{i}\Delta {\bf{W}}(t)$$

to track all the 20 quadrature expectations at different times. Here, $${A}_{i}^{{\prime} }=\left[\begin{array}{cc}-{\varGamma }_{{\rm{m}}}^{{\prime} i}\,/2 & {\varOmega }_{i}^{{\prime} }-{\varOmega }_{{\rm{m}}}\\ {\varOmega }_{{\rm{m}}}-{\varOmega }_{i}^{{\prime} } & -{\varGamma }_{{\rm{m}}}^{{\prime} i}\,/2\end{array}\right]$$ contains modified mechanical parameters:

$$\begin{array}{l}{\varGamma }_{{\rm{m}}}^{{\prime} i}={\varGamma }_{{\rm{m}}}^{i}+2{\rm{Re}}\,\left[-\frac{1-\cos (({\varOmega }_{i}-{\varOmega }_{{\rm{m}}})\Delta t)}{\Delta t}\right]\\ {\varOmega }_{i}^{{\prime} }={\varOmega }_{i}-{\rm{Im}}\,\left[i({\varOmega }_{i}-{\varOmega }_{{\rm{m}}})-\frac{{{\rm{e}}}^{{\rm{i}}({\varOmega }_{i}-{\varOmega }_{{\rm{m}}})\Delta t}-1}{\Delta t}\right]\end{array}$$

to compensate for the influence of discretization on the state estimation performance compared with an ideal continuous one.

The evolution of the matrix *B*_*i*_, involving 210 independent covariance matrix elements, can be computed independently from the sampled time-domain data. Therefore, we calculate it following equation (3), with an update rate of 140 MHz to mitigate the discretization effect, which is then used for the update equation (4) at the sampling rate of 14 MHz. The verification of the correct implementation of the multimode Kalman filter is shown in the Supplementary Information.

To experimentally reconstruct the covariance matrix from the estimated quadrature data, we use the retrodiction method. The retrodiction method uses the measurement record in the future as a separate state estimation result. We derived the retrodiction update equations^39^ and found that they are identical to the prediction update equations, except with negative mechanical frequencies. As a result, we have the following relations between covariance matrix elements estimated by prediction and retrodiction (respectively identified by the superscripts p and r):

$$\begin{array}{l}{C}_{{\widehat{X}}_{i}{\widehat{X}}_{j}}^{{\rm{p}}}={C}_{{\widehat{X}}_{i}{\widehat{X}}_{j}}^{{\rm{r}}}\\ \,{C}_{{\widehat{Y}}_{i}{\widehat{Y}}_{j}}^{{\rm{p}}}={C}_{{\widehat{Y}}_{i}{\widehat{Y}}_{j}}^{{\rm{r}}}\\ {C}_{{\widehat{X}}_{i}{\widehat{Y}}_{j}}^{{\rm{p}}}=-{C}_{{\widehat{X}}_{i}{\widehat{Y}}_{j}}^{{\rm{r}}}.\end{array}$$

For each time trace slice (1 ms), we calculate the difference between the prediction and retrodiction results ${\langle \widehat{{\bf{r}}}\rangle }_{{\rm{r}}}-{\langle \widehat{{\bf{r}}}\rangle }_{{\rm{p}}}$, and calculate the covariance matrix as

$$C=\frac{1}{2}\langle \langle \left({\langle \widehat{{\bf{r}}}\rangle }_{{\rm{r}}}-{\langle \widehat{{\bf{r}}}\rangle }_{{\rm{p}}}\right)\cdot {\left({\langle \widehat{{\bf{r}}}\rangle }_{{\rm{r}}}-{\langle \widehat{{\bf{r}}}\rangle }_{{\rm{p}}}\right)}^{\top }\rangle \rangle $$

where ⟨⟨⋯⟩⟩ is the statistical average over all the time trace slices, and $\widehat{{\bf{r}}}=\left[\cdots ,{\widehat{X}}_{i},{\widehat{Y}}_{i},\cdots \right]$. The symbol^T^ indicates the transposed vector.

For a system consisting of several mechanical modes that are not sufficiently separated in frequency (∣*Ω*_*i*_ − *Ω*_*j*_∣ not significantly faster than any other rates in the system), cross-correlations between different mechanical modes emerge because of common measurement imprecision noise and common quantum backaction force. This generally leads to higher quadrature variance because of the effectively reduced measurement efficiency of individual modes. To decouple the mechanical oscillators that are interacting because of the spectral overlap and the measurement process, we define a new set of collective motional modes through a symplectic (canonical) transformation of quadrature basis *U* that diagonalizes the covariance matrix *U*^†^*CU* = *V* (ref. ^55^). As the covariance matrix is real and symmetric, the elements of *U* are always real, which is required for real observables. The transformation can be understood as a normal mode decomposition of the collective Gaussian state that preserves the commutation relations, as opposed to conventional diagonalization using unitary matrices. This is represented by the requirement of the symplectic transformation *UΩU*^†^ = *Ω*, where $$\varOmega =\left[\begin{array}{cc}0 & {I}_{N}\\ -{I}_{N} & 0\end{array}\right]$$ is the N-mode symplectic form and *I*_*N*_ is the *N* × *N* identity matrix. We find that in the new quadrature basis based on the diagonalized covariance matrix, the defect mode is only weakly modified. The transformation coefficients for the defect mode are shown in the Supplementary Information.

## Online content

Any methods, additional references, Nature Portfolio reporting summaries, source data, extended data, supplementary information, acknowledgements, peer review information; details of author contributions and competing interests; and statements of data and code availability are available at 10.1038/s41586-023-06997-3.

### Supplementary information

Supplementary Information Supplementary Sections A–K, including Supplementary Figures, Tables, Text and data – see Contents for details.

## Data Availability

The code and data used to produce the plots in this work are available on the Zenodo repository (10.5281/zenodo.10040032).
