# Supplementary material for: Room-temperature quantum optomechanics using an ultralow noise cavity
Source: Nature. 2024 Feb 14;626(7999):512–6. doi: 10.1038/s41586-023-06997-3 (PMC10866701; doi:10.1038/s41586-023-06997-3)
Supplement: Supplementary file 1 — Supplementary Sections A–K, including Supplementary Figures, Tables, Text and data – see Contents for details. [file 41586_2023_6997_MOESM1_ESM.pdf]

nature portfolio

https://doi.org/10.1038/s41586-023-06997-3

Supplementary information

Room-temperature quantum optomechanics using an ultralow noise cavity

In the format provided by the authors and unedited

Supplementary Information for: "Room-temperature quantum optomechanics using an ultra-low noise cavity"

Guanhao Huang,[1,2,*] Alberto Beccari,[1,2,*] Nils J. Engelsen,[1,2,3,†] and Tobias J. Kippenberg[1,2,‡]

[1] *Swiss Federal Institute of Technology Lausanne (EPFL), CH-1015 Lausanne, Switzerland*
[2] *Center for Quantum Science and Engineering, EPFL, CH-1015 Lausanne, Switzerland*
[3] *Department of Microtechnology and Nanoscience (MC2), Chalmers University of Technology, SE-412 96 Göteborg, Sweden*

CONTENTS

A. System parameters & calibration 1

B. Signal shot noise calibration 3

C. Elimination of TIN in photodetection 3

D. Quantum Langevin equations of the optomechanical system 5

E. Multimode state estimation model 7

F. Numerical implementation of multimode state estimation 8

G. Sideband cooling limit 9

H. Mechanical simulations of density-modulated PnC membranes 10

I. Microfabrication of density-modulated PnC membranes 13

J. Fabrication and simulation of phononic crystal mirrors 15

K. TiSa laser phase noise characterization 16

References 16

Section A: System parameters & calibration

We characterized several experimental parameters of the optomechanical cavity prior to the measurements shown in the manuscript and use them as pre-determined parameters for the fits of the measured mechanical spectra. We obtained cavity resonance linewidths κ at different optical wavelengths, shown in Fig. 2(b), by scanning our Titanium:Sapphire (TiSa) laser frequency at a rate of 300 MHz/s across the optical resonances, with optical power smaller than 1 µW to prevent significant mechanical excitation when the laser frequency crosses to the blue side of the cavity resonance [1]. The laser is phase modulated at 211 MHz with modulation depth ~ 0.1 to generate two small sidebands, which serve as the reference points for calibrating the frequency axis. A Lorentzian function is fitted to the cavity transmission to extract the optical linewidth.

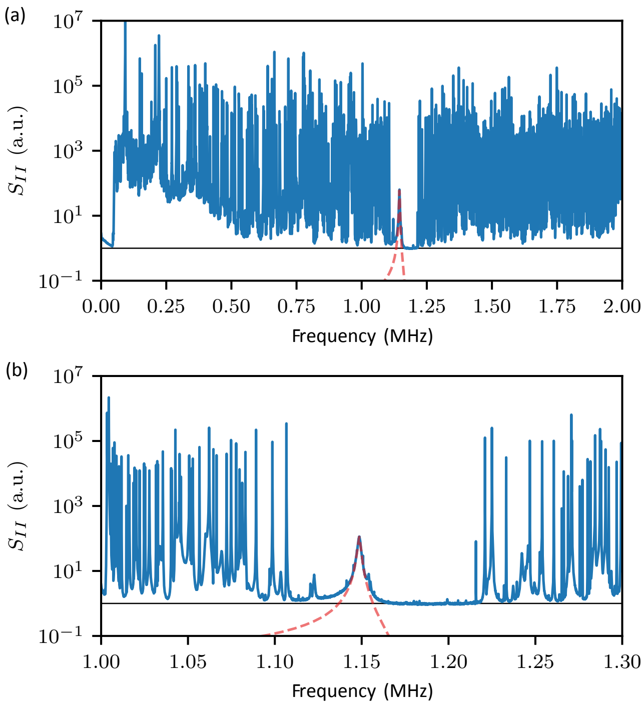

FIG. A.1. Mechanical spectra in **(a)** wideband and **(b)** narrowband frequency spans, measured from the optical direct detection of the cavity transmission beam. The cavity is pumped at the magic detuning on the red side, enacting significant optical damping on all the mechanical modes. The high-Q defect-mode signal after shot noise (indicated by the black line) subtraction is highlighted in red dashed line, whose asymmetric spectral shape is an indication of strong quantum correlation with the measurement shot noise.

The mechanical spectral properties of the MIM system is characterized by the optical direct detection of the cavity thermal mechanical noises from the transmission beam. As illustrated in Fig. A.1, the thermal mechanical noise is dominated by the densely packed membrane modes. The membrane spectra exhibit a 100-kHz mechanical bandgap between 1.1 MHz and 1.2 MHz as a result of the effective periodic density modulation induced

* These authors contributed equally to this work.
† nils.engelsen@chalmers.se
‡ tobias.kippenberg@epfl.ch

by the nano pillars. A defect mode is localized in the center of the bandgap. The mechanical frequency of the membrane defect mode, Ω_m, is measured from the cavity transmission spectrum at low input optical power, to limit optical spring effects due to dynamical backaction. The mechanical energy decay rate Γ_m is measured with a mechanical ringdown measurement (see Fig. 1(d)). In the ringdown experiment, a pump laser is first tuned to the blue side of an optical cavity mode at 845 nm (high finesse) to strongly excite the membrane defect mode (phonon lasing), then the pump laser is switched off and a weak probe laser is switched on near a cavity mode at 780 nm (low finesse) to measure the mechanical oscillation amplitude decay through the cavity transmission signal. The same ringdown measurement is repeated at multiple probe laser detuning frequencies, to ensure no dynamical backaction damping or amplification is present during the ringdown measurement. The extracted damping rate is consistent with a previous measurement of the membrane in an optical interferometer, before cavity assembly, indicating that no significant mechanical dissipation is added to the defect mode during the MIM assembly procedure.

To obtain vacuum optomechanical coupling rates, g_0, we follow the procedure of [2]. We phase-modulate a weak laser probe with known depth, and compare the mechanical signal to the phase modulation tone transduced on the transmitted light intensity, directly detected at the cavity output. The probe laser is stabilized at the magic detuning to avoid heating from intracavity TIN. We assume the mechanical mode is initially thermalized to the room temperature environment, and undergoes dynamical backaction cooling by the red-detuned probe laser. The probe power is kept relatively low ($\mathcal{C}_q \ll 1$) to avoid quantum backaction heating, such that the thermal occupation of the defect mode can be reliably inferred from the dynamical backaction cooling rate found by fitting the mechanical spectrum. The probe laser is phase modulated at 1.1457 MHz with a depth of 0.1275, and at 1.19 MHz with a depth of 0.152. The two modulation frequencies are selected such that they are at the edges of the mechanical bandgap, and are symmetric with respect to the frequency of the defect mode. The geometric average of their transduced signals is calculated in order to cancel the OMIT frequency response [3] from the defect mode and obtain an estimate of g_0. In the actual experiment, however, the field enhanced optomechanical coupling rate $g = g_0\overline{a}$ is the relevant parameter, where $\overline{a}$ is the cavity field amplitude. g is left as a free parameter in all the fits of the measured mechanical spectra. In the experiments shown in the main text, where $\mathcal{C}_q \sim 1$, we have $g/2\pi \sim$500 kHz.

The aforementioned system parameters are summarized in the table below.

System parameter	Value
$\Omega_m/2\pi$	1.167 MHz
$g_0/2\pi$	159.0 Hz
$\kappa/2\pi$	34.2 MHz
$\Gamma_m/2\pi$	6.41 mHz
C_0	0.461

We now discuss the various contributions to the detection efficiency η_d, which is an essential figure of merit for the state preparation experiment. In the experiments, we retrieve the detection efficiency by spectral fitting of the squeezing/mechanical signal, which depends sensitively on it. In the following, we individually identify and describe the calibration of some of the inefficiency contributions in the measurement chain:

- The detector electronics noise is measured by blocking the optical beam. The electronics noise is flat and broadband, so the inefficiency contribution from it can be retrieved by comparing it to the laser shot noise.

- The transmission from the optical elements after the cavity output and before the detector is measured with a handheld power meter. This loss term is dominated by a series of polarizing beam splitters, a beam pick-off for generating an error signal for laser locking, several waveplates, and the highly asymmetric beam splitter which combines the local oscillator with the signal beam.

- The detector quantum efficiency is calculated from the detector responsivity, specified by the manufacturer.

- The homodyne efficiency is calculated from the mode matching efficiency of 90% between the local oscillator and signal beam, given the measured interference visibility of 95%. The efficiency is only 75% in contrast to the usual 90% in conventional balanced homodyne detection, due to the fact that we are using a single detector homodyne scheme to cancel the nonlinear mixing noise. The single detector homodyne has a quadrature angle-dependent efficiency: the efficiency is lower when measuring mechanical motion, and higher for optomechanical squeezing detection. The quadrature angle-dependent homodyne efficiencies are shown in Fig. C.1(d).

- The cavity output efficiency is hard to infer from the cavity reflection scan due to the unknown optical input mode-matching efficiency, and from the empty cavity characterization due to the redistributed intra-cavity optical power in the presence of the weakly reflective membrane. We assume a perfect cavity out-coupling efficiency of 94.8%, and consider the added cavity inefficiency from membrane scattering and absorption as part of the overall detection inefficiency in the measurement chain.

This residual inefficiency is retrieved from the spectral fitting of the mechanical signal.

- The defect-mode conversion to mixing sidebands at other frequencies due to the cavity transduction nonlinearity could reduce the linear transduction efficiency as well. It is in principle also included in the fitted residual inefficiency. From the measured $\langle \delta\Delta^2 \rangle / \kappa^2 < 10^{-3}$, this contribution is estimated to be less than 2%, and thus excluded from the analysis.

The various inefficiency terms degrading the SNR of the mechanical displacement records are shown in the table below.

Inefficiency source	Value
Cavity output (ideal)	94.8 %
Detector electronics noise	86.1 %
Passive optics	66.5 %
Detector quantum efficiency	90 %
Homodyne efficiency	75 %
Fitted additional loss	83.5 %
**Total efficiency**	30.6 %

Section B: Signal shot noise calibration

To calibrate the reference shot noise power spectral density for a given signal, we proceed as follows:

1. Record the average detector voltage output during data acquisition for each optical power level impinging on the detector,

2. Block the beam from the output of the optical cavity such that only the local oscillator beam hits the detector,

3. Adjust the local oscillator power such that the average detector voltage output is the same as the one recorded during the data acquisition. This can be done with accuracy better than 1 %,

4. Record the noise power spectral density of the detector voltage output through the same electrical signal chain as the data acquisition, which serves as the reference shot noise power spectral density.

Since the detector voltage output is the same, the generated photocurrent flux and the optical noise power should be the same as well. However, it must be verified that the local oscillator beam is shot-noise-limited at the Fourier frequency of interest. The TiSa laser we use is predominantly shot-noise-limited at around 1 MHz under our experimental conditions. We quantify this by recording the photocurrent noise scaling as the optical power is varied. The laser shot noise scales linearly with the optical power, which is proportional to the detector DC voltage output, while any classical noise contribution scales quadratically. We fit the local oscillator noise power integrated over each frequency band as a function of detector DC voltage with a polynomial function consisting only the 1st-order and the 2nd-order terms, as shown in Fig. B.1(a). We confirm that within the frequency range of interest and optical powers used in the experiment, the classical noise contribution is less than 1 %, as shown in Fig. B.1(b). Using the fit results, we correct the measured reference shot noise by subtracting the calibrated classical noise contribution to ensure that we do not overstate the amount of squeezing. We also measured the detector DC voltage output linearity with respect to optical power with a reference power meter, and found a maximum deviation of less than 0.3 %.

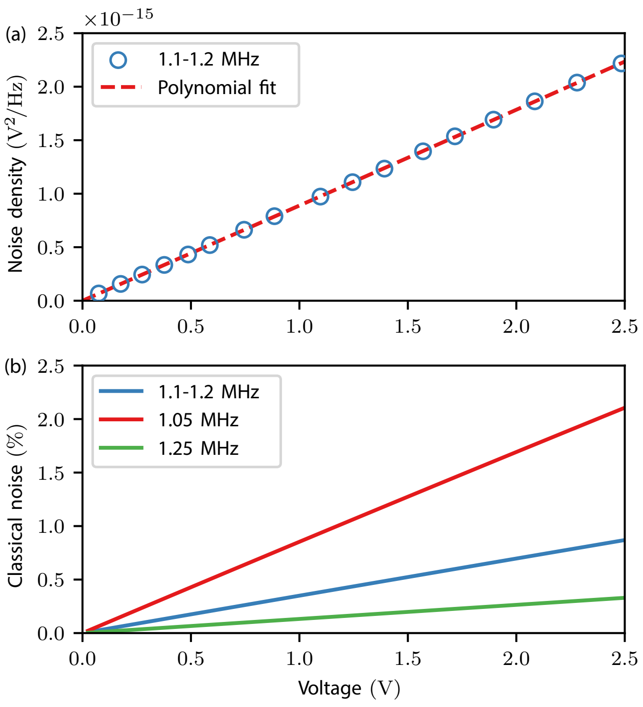

FIG. B.1. **(a)** Laser intensity noise power (detector noise subtracted) averaged over 1.1-1.2 MHz frequency band, as a function of detector output voltage. A polynomial function consisting of linear and quadratic terms is used to fit and extract the contribution of the laser classical noise. **(b)** The laser classical noise fractional contribution to the intensity noise is plotted as a function of detector voltage, over three frequency bands. Most of the classical noise is the high frequency tail from the laser relaxation oscillation peak around 300 kHz. At the frequency band of interest 1.1-1.2 MHz where the experiment is conducted, the classical noise contribution is less than 1 %.

Section C: Elimination of TIN in photodetection

The thermal intermodulation noise from the nonlinear cavity transduction response can be derived from the classical Langevin equation for the evolution of the cavity

field:

$$\dot{a}(t) = [i(\overline{\Delta} + \delta\Delta(t) - \kappa/2]a(t) + \sum_i \sqrt{\kappa_i} a_{\mathrm{in},i}(t) \tag{C1}$$

where $a_{\mathrm{in},i}$ are the different coupling channels of the cavity (including the intracavity losses), $\overline{\Delta}$ is the mean value of the laser-cavity detuning, and the $\delta\Delta(t)$ term includes all cavity frequency fluctuations, such as those due to membrane motion and mirror vibrations. The frequency domain equivalent is a Fredholm integral equation of the second kind, whose solution can be written as a Liouville-Neumann series of different orders, $a(\omega) = \sum_n a_n(\omega)$. The 0th order includes the DC response and vacuum fluctuations. We are mostly concerned with the 1st and 2nd orders, that provide the linear and quadratic transduction of mechanical motion:

$$a_1(\omega) = \overline{a}\frac{i\Delta(\omega)}{i(-\omega - \overline{\Delta}) + \kappa/2}, \tag{C2}$$

$$a_2(\omega) = -\overline{a}\int \frac{\Delta(\omega-\omega')}{i(-\omega - \overline{\Delta}) + \kappa/2}\frac{\Delta(\omega')}{i(-\omega' - \overline{\Delta}) + \kappa/2}\frac{d\omega'}{2\pi}, \tag{C3}$$

where $\overline{a}$ is the mean cavity field, $\overline{\Delta}$ is the mean laser detuning, and $\Delta(\omega) = \mathrm{FT}[\delta\Delta(t)]$ is dominated by the cavity thermal mechanical noise (FT indicates a Fourier transform).

The intracavity photon number noise is given by:

$$n_{\mathrm{cav}}(\omega) = \overline{a}^* \left[a_1(\omega) + a_2(\omega)\right] + h.c. \tag{C4}$$

$$+\int \frac{d\omega'}{2\pi} a_1^\dagger(\omega-\omega')a_1(\omega'), \tag{C5}$$

and exhibits mixing noise terms both from the cavity nonlinear transduction, $a_2(\omega)$, and the intrinsic nonlinearity of the photon number operator, $\mathrm{FT}\left[a_1^\dagger(t)a_1(t)\right]$. The $h.c.$ symbol indicates the hermitian conjugate of the previous term. In the bad-cavity limit ($\omega \ll \kappa$), we find that at the magic detuning, $2\overline{\Delta}/\kappa = -1/\sqrt{3}$, the nonlinear photon number noise is cancelled, and the conventional linear transduction $n_{\mathrm{cav}}(\omega) = \overline{a}^* a_1(\omega) + h.c.$ is kept. Operation at the magic detuning prevents excess oscillator heating due to nonlinear classical radiation pressure noise.

In order to reveal the quantum correlations leading to optomechanical squeezing, we need to perform measurements at arbitrary optical quadrature angles. Balanced homodyne detection provides the possibility of tuning the optical quadrature, but does not offer enough degrees of freedom to cancel the nonlinear noise at arbitrary quadrature angles. The photocurrent signal in balanced homodyne detection is given by:

$$\Delta I = I_+ - I_- \propto \overline{a}_{\mathrm{LO}}^* \left[a_1(\omega) + a_2(\omega)\right] + h.c., \tag{C6}$$

where $\overline{a}_{\mathrm{LO}}$ is the local oscillator (LO) field amplitude. The field nonlinearity $a_2(\omega)$ is generally non-zero in this expression. However, if the LO is injected from a highly asymmetric beam splitter with a very small reflectivity ($r \ll 1$) and the combined field is detected on a single photodiode, the photocurrent acquires another quadratic term:

$$I \propto \overline{a}_{\mathrm{hom}}^* \left[a_1(\omega) + a_2(\omega)\right] + h.c. \tag{C7}$$

$$-\sqrt{\kappa_{\mathrm{ex}}}\int \frac{d\omega'}{2\pi} a_1^\dagger(\omega-\omega')a_1(\omega'), \tag{C8}$$

where $\kappa_{\mathrm{ex}} = \kappa_a$ is the cavity output coupling rate. In this case, simultaneous tuning of LO amplitude and phase enables nonlinear mixing noise cancellation at arbitrary quadrature angles. In the bad-cavity limit, the cancellation condition is:

$$\begin{aligned}\left|\frac{\overline{a}_{\mathrm{sig}}}{\overline{a}_{\mathrm{hom}}}\right| &= 2\mathrm{Re}\left[\frac{e^{-i\theta}}{(-i\overline{\Delta} + \kappa/2)^2}\right]\left[\overline{\Delta}^2 + \left(\frac{\kappa}{2}\right)^2\right] \\ &= 2\cos\left[\theta - 2\arg\left(\chi_{\mathrm{cav}}(0)\right)\right],\end{aligned} \tag{C9}$$

where $\overline{a}_{\mathrm{hom}} \approx \overline{a}_{\mathrm{sig}} + r\overline{a}_{\mathrm{LO}}$ is the coherent combination of the signal field $\overline{a}_{\mathrm{sig}} = -\sqrt{\kappa_{\mathrm{ex}}}\overline{a}$ and the LO field $\overline{a}_{\mathrm{LO}}$ (defined as the amplitude before the beam splitter), and $\theta = \theta_{\mathrm{hom}} - \theta_{\mathrm{sig}}$ is the quadrature rotation angle (cfr. phasor diagram in Fig. 3). $\chi_{\mathrm{cav}}(0) = \left(\kappa/2 - i\overline{\Delta}\right)^{-1}$ is the cavity DC optical susceptibility. We show the required local oscillator intensity $I_{\mathrm{LO}} = |r\overline{a}_{\mathrm{LO}}|^2$ and the combined field intensity $I_{\mathrm{hom}} = |\overline{a}_{\mathrm{hom}}|^2$ in order to cancel the nonlinear noise as a function of quadrature angle θ in Fig. C.1(c).

In the experiment, to detect a certain quadrature angle while cancelling nonlinear noise, we lock the homodyne power at the corresponding combined field intensity I_{hom}. Then, we continuously vary the local oscillator power using a tunable neutral density filter until the noise in the mechanical bandgap is perfectly cancelled, as is shown in Fig. C.1(a). The level of mixing noise is very sensitive to the local oscillator power, and therefore the cancellation point can serve as a good indicator of the measured quadrature angle θ. Knowing the field amplitude ratios between $|\overline{a}_{\mathrm{hom}}|$, $|\overline{a}_{\mathrm{sig}}|$, and that $\overline{\Delta} = -\kappa/(2\sqrt{3})$, we can reconstruct the measured quadrature angles as the ones satisfying the condition in C9. The nonlinear noise level in a frequency band inside the mechanical bandgap as a function of local oscillator power is shown in Fig. C.1(b).

The detection efficiency in this setting is different from the balanced homodyne case, where it is given by the square of the interference visibility v between the signal beam and the LO beam. In our case, the LO and signal beams have comparable optical powers. The added noise comes from the mode-mismatched LO intensity $\Delta I_{\mathrm{LO}} = |r\overline{a}_{\mathrm{LO}}|^2(1/v^2 - 1)$, which results in the reduced homodyne efficiency of $\eta_{\mathrm{hom}} = I_{\mathrm{hom}}/(I_{\mathrm{hom}} + \Delta I_{\mathrm{LO}})$. Since the local oscillator power is determined by the requirement of nonlinear noise elimination (equation C9), the homodyne efficiency acquires a quadrature angle dependence. We plot the efficiency at different quadrature angles in Fig. C.1(d) given the experimentally characterized visibility $v = 95\%$.

Note that the nonlinear mixing noise can be perfectly cancelled only in the bad cavity limit. In reality, the level

of cancellation is limited by three factors. The first is the laser detuning stability, the second is the homodyne angle stability, and the third is the partial breakdown of the bad cavity limit due to the finite cavity bandwidth ($\kappa/\Omega_m \approx 30$ in our experiment). The finite ratio results in a non-negligible noise floor for various system observables, e.g. for the intracavity photon number:

$$S_{n_{\text{cav}}n_{\text{cav}}}(\omega) \propto \int S_{\Delta\Delta}(\omega-\omega')S_{\Delta\Delta}(\omega')d\omega'$$
$$\times \frac{(4\overline{\Delta}^2+\kappa^2)(-12\overline{\Delta}^2+\kappa^2)^2+8(-4\overline{\Delta}^2+3\kappa^2)\kappa^2\omega^2}{(4\overline{\Delta}^2+\kappa^2)^5}$$

At low optical power (output field on the order of 10 µW) accompanied with low passive optical cooling, we find that the combination of these effects limits the cancellation factor at magic detuning to about 300 for the intracavity photon number noise measured with direct detection. At high optical powers, as employed for the experiment, the passive optical cooling is strong enough that this noise floor is hardly visible in detection.

Section D: Quantum Langevin equations of the optomechanical system

Here, we derive the measurement noise spectral density expression that is used for fitting the experimental data. In the Heisenberg picture, the quantum Langevin equations are:

$$\dot{\hat{a}} = -\left[\frac{\kappa}{2} + i(\Delta + \sqrt{2}g_0\hat{Q})\right]\hat{a} + \sum_{i=a,b,\cdots}\sqrt{\kappa_i}\hat{a}_{\text{in,i}},$$

for the cavity mode annihilation operator $\hat{a}$ and input mode operators $\hat{a}_{\text{in,i}}$, where the detuning is defined as $\Delta = \omega_{\text{cav}} - \omega_{\text{laser}}$. For the mechanical mode evolution:

$$\dot{\hat{Q}} = \Omega_m\hat{P}$$
$$\dot{\hat{P}} = -\Omega_m\hat{Q} + \sqrt{2\Gamma_m}\hat{P}_{\text{in}} - \Gamma_m\hat{P} - \sqrt{2}g_0\hat{a}^\dagger\hat{a},$$

where we defined the dimensionless mechanical position operator $\hat{Q} = (\hat{b}^\dagger + \hat{b})/\sqrt{2}$ and momentum operator $\hat{P} = i(\hat{b}^\dagger - \hat{b})/\sqrt{2}$; $\hat{b}$ is the mechanical mode's annihilation operator, and $\hat{P}_{\text{in}}(t) = \frac{i}{\sqrt{2}}\left(\hat{b}^\dagger_{\text{in}}(t) - \hat{b}_{\text{in}}(t)\right)$ is the input momentum fluctuation. We can also define the optical quadrature operators $\hat{X} = (\hat{a}^\dagger + \hat{a})/\sqrt{2}$ and $\hat{Y} = i(\hat{a}^\dagger - \hat{a})/\sqrt{2}$. Note that these quadrature definitions are only used within this section. It is also convenient to define the optomechanical coupling rate $g = g_0\overline{a}$. Combining the evolution equations for the quadratures, we obtain for the mechanical position operator:

$$\ddot{\hat{Q}} + \Gamma_m\dot{\hat{Q}} + \Omega_m^2\hat{Q} = \sqrt{2\Gamma_m}\Omega_m\hat{P}_{\text{in}} - 2g\Omega_m\left(\hat{X} + X_{\text{nl}}\right)$$
$$\hat{Q}(\omega) = \chi_m(\omega)\left(\sqrt{2\Gamma_m}\hat{P}_{\text{in}}(\omega) - 2g\hat{X}(\omega)\right),$$

where we introduced the frequency-domain mechanical susceptibility $\chi_m(\omega) \equiv \Omega_m/(\Omega_m^2 - \omega^2 - i\omega\Gamma_m)$, the nonlinear noise X_{nl}, which is cancelled at the magic detuning

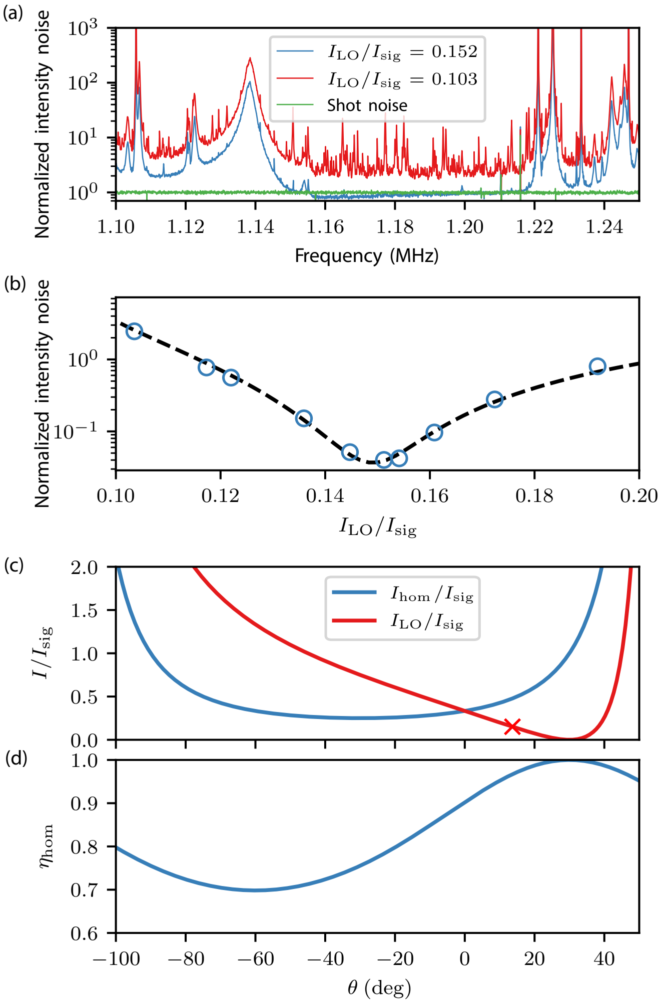

FIG. C.1. **(a)** When the homodyne power is locked to $I_{\text{hom}}/I_{\text{sig}} = 0.481$, different injected local oscillator intensities I_{LO} result in different optical quadratures being probed, and drastically different nonlinear noise levels in detection. When the appropriate local oscillator intensity is injected, the nonlinear noise can be efficiently cancelled (blue). **(b)** The averaged nonlinear noise from 1.16 MHz to 1.18 MHz, normalized to shot noise, is plotted as a function of local oscillator intensity I_{LO} (theory fit in dashed line, including a 4 % noise offset from nearby mechanical modes). **(c)** Required I_{hom} and I_{LO} for nonlinear noise cancellation at different quadrature angles θ. The angles are displayed after subtraction of the the cavity-induced angle rotation of −30° at the magic detuning. The red cross marks $I_{\text{LO}}/I_{\text{sig}} = 0.150$, the noise cancellation condition shown in **(a,b)**. **(d)** Homodyne detection efficiency at different quadrature angles, given the experimentally characterized $v = 0.95$.

(and set to zero for the following derivations). The dimensionless input momentum fluctuation $\hat{P}_{\text{in}}(t)$ is characterized by the following spectral correlations:

$$S_{P_{\text{in}}P_{\text{in}}}(\omega) = \frac{\omega}{\Omega_m}(\overline{n}_{\text{th}}(\omega)+1)$$
$$S_{P_{\text{in}}P_{\text{in}}}(-\omega) = \frac{\omega}{\Omega_m}\overline{n}_{\text{th}}(\omega)$$

Now for simplicity, the mean cavity field $\overline{a}$ is used as the phase reference $\theta = 0$ for other fields, such that both $\overline{a}$ and g are real valued. Considering all optical channels $\hat{a}_{\mathrm{in,i}}$, we define the following susceptibilities:

$$\begin{aligned}
\chi_c(\omega) &= 2^{-1/2}(\kappa/2 + i\Delta - i\omega)^{-1} \\
\chi_c^X(\omega) &= i(\chi_c^*(-\omega) - \chi_c(\omega)) \\
\chi_c^Y(\omega) &= -(\chi_c^*(-\omega) + \chi_c(\omega)) \\
\chi_{mc}^X(\omega) &= (1 + 2\sqrt{2}g^2\chi_m(\omega)\chi_c^X(\omega))^{-1} \\
\chi_\Delta^X(\omega) &= \overline{a}\chi_c^X(\omega)\chi_{mc}^X(\omega)
\end{aligned}$$

$$\begin{aligned}
\chi_{P_{\mathrm{in}}}^X(\omega) &= \sqrt{2}g\chi_m(\omega)\chi_c^X(\omega)\chi_{mc}^X(\omega) \\
\chi_{a_{\mathrm{in,i}}}^X(\omega) &= \sqrt{\kappa_i}\chi_c(\omega)\chi_{mc}^X(\omega) \\
\chi_{a_{\mathrm{in,i}}^\dagger}^X(\omega) &= \sqrt{\kappa_i}\chi_c^*(-\omega)\chi_{mc}^X(\omega) \\
\chi_\Delta^Y(\omega) &= \chi_c^Y(\omega)[\overline{a} - 2\sqrt{2}g^2\chi_m(\omega)\chi_\Delta^X(\omega)] \\
\chi_{P_{\mathrm{in}}}^Y(\omega) &= \sqrt{2}g\chi_c^Y(\omega)\chi_m(\omega)(1 - 2g\chi_{P_{\mathrm{in}}}^X(\omega)) \\
\chi_{a_{\mathrm{in,i}}}^Y(\omega) &= -i\sqrt{\kappa_i}\chi_c(\omega) - 2\sqrt{2}g^2\chi_c^Y(\omega)\chi_m(\omega)\chi_{a_{\mathrm{in,i}}}^X(\omega) \\
\chi_{a_{\mathrm{in,i}}^\dagger}^Y(\omega) &= i\sqrt{\kappa_i}\chi_c^*(-\omega) - 2\sqrt{2}g^2\chi_c^Y(\omega)\chi_m(\omega)\chi_{a_{\mathrm{in,i}}^\dagger}^X(\omega)
\end{aligned}$$

We obtain the following frequency domain equations for the optical and mechanical quadratures:

$$\begin{aligned}
\hat{X}(\omega) &= \chi_\Delta^X(\omega)\Delta(\omega) + \chi_{P_{\mathrm{in}}}^X(\omega)\sqrt{2\Gamma}\hat{P}_{\mathrm{in}}(\omega) + \sum_{i=a,b,\ldots}\left(\chi_{a_{\mathrm{in,i}}}^X(\omega)\hat{a}_{\mathrm{in,i}}(\omega) + \chi_{a_{\mathrm{in,i}}^\dagger}^X(\omega)\hat{a}_{\mathrm{in,i}}^\dagger(\omega)\right) \\
\hat{Y}(\omega) &= \chi_\Delta^Y(\omega)\Delta(\omega) + \chi_{P_{\mathrm{in}}}^Y(\omega)\sqrt{2\Gamma}\hat{P}_{\mathrm{in}}(\omega) + \sum_{i=a,b,\ldots}\left(\chi_{a_{\mathrm{in,i}}}^Y(\omega)\hat{a}_{\mathrm{in,i}}(\omega) + \chi_{a_{\mathrm{in,i}}^\dagger}^Y(\omega)\hat{a}_{\mathrm{in,i}}^\dagger(\omega)\right) \\
\hat{Q}(\omega) &= \chi_m(\omega)\left\{(1 - 2g\chi_{P_{\mathrm{in}}}^X)\sqrt{2\Gamma}\hat{P}_{\mathrm{in}}(\omega) - 2g\left[\chi_\Delta^X\Delta(\omega) + \sum_{i=a,b,\ldots}\left(\chi_{a_{\mathrm{in,i}}}^X(\omega)\hat{a}_{\mathrm{in,i}}(\omega) + \chi_{a_{\mathrm{in,i}}^\dagger}^X(\omega)\hat{a}_{\mathrm{in,i}}^\dagger(\omega)\right)\right]\right\}
\end{aligned}$$

We also define quadratures with arbitrary angles $\hat{X}^\theta = 2^{-1/2}(\hat{a}e^{-i\theta} + \hat{a}^\dagger e^{i\theta}) = \hat{X}\cos\theta + \hat{Y}\sin\theta$. We pick $\hat{a}_{\mathrm{in,a}}$ to be the cavity output port, with $\hat{a}_{\mathrm{in,b}}$, $\hat{a}_{\mathrm{in,c}}$ the input and loss channels. Here, we consider the output field from the transmission port of the cavity, which combines both the cavity output field and the input vacuum field reflection at the transmission port). From the input-output formalism, the output quadrature is:

$$\hat{X}_{\mathrm{out}}^\theta = \hat{X}_{\mathrm{in,a}}^\theta - \sqrt{\kappa_a}\hat{X}^\theta.$$

With the following susceptibilities ($k = b, c$, Fourier frequency dependence omitted):

$$\begin{aligned}
\chi_\Delta^\theta &= -\sqrt{\kappa_a}(\chi_\Delta^X\cos\theta + \chi_\Delta^Y\sin\theta) \\
\chi_{P_{\mathrm{in}}}^\theta &= -\sqrt{\kappa_a}(\chi_{P_{\mathrm{in}}}^X\cos\theta + \chi_{P_{\mathrm{in}}}^Y\sin\theta)
\end{aligned}$$

$$\begin{aligned}
\chi_{a_{\mathrm{in,a}}}^\theta &= \sqrt{2}^{-1}e^{-i\theta} - \sqrt{\kappa_a}(\chi_{a_{\mathrm{in,a}}}^X\cos\theta + \chi_{a_{\mathrm{in,a}}}^Y\sin\theta) \\
\chi_{a_{\mathrm{in,a}}^\dagger}^\theta &= \sqrt{2}^{-1}e^{i\theta} - \sqrt{\kappa_a}(\chi_{a_{\mathrm{in,a}}^\dagger}^X\cos\theta + \chi_{a_{\mathrm{in,a}}^\dagger}^Y\sin\theta) \\
\chi_{a_{\mathrm{in,k}}}^\theta &= -\sqrt{\kappa_k}(\chi_{a_{\mathrm{in,k}}}^X\cos\theta + \chi_{a_{\mathrm{in,k}}}^Y\sin\theta) \\
\chi_{a_{\mathrm{in,k}}^\dagger}^\theta &= -\sqrt{\kappa_k}(\chi_{a_{\mathrm{in,k}}^\dagger}^X\cos\theta + \chi_{a_{\mathrm{in,k}}^\dagger}^Y\sin\theta)
\end{aligned}$$

and taking into account photon collection efficiency η, the detected optical quadrature is expressed as:

$$\hat{X}_{\mathrm{det}}^\theta(\omega) = \sqrt{\eta}\left[\chi_\Delta^\theta\Delta(\omega) + \chi_{P_{\mathrm{in}}}^\theta\sqrt{2\Gamma}\hat{P}_{\mathrm{in}}(\omega) + \sum_{i=a,b,c}\left(\chi_{a_{\mathrm{in,i}}}^\theta\hat{a}_{\mathrm{in,i}}(\omega) + \chi_{a_{\mathrm{in,i}}^\dagger}^\theta\hat{a}_{\mathrm{in,i}}^\dagger(\omega)\right)\right] + i\sqrt{1-\eta}\hat{X}_{\mathrm{vac}}(\omega).$$

Using the following correlation relations,

$$S_{AB}(\omega) = \int\left\langle A^\dagger(\omega)B(\omega')\right\rangle\frac{d\omega'}{2\pi}$$

$$\begin{aligned}
\left\langle a_{\mathrm{in}}(\omega)a_{\mathrm{in}}^\dagger(\omega')\right\rangle &= 2\pi\delta(\omega + \omega') \\
\left\langle a_{\mathrm{in}}^\dagger(\omega)a_{\mathrm{in}}(\omega')\right\rangle &= 0,
\end{aligned}$$

we can calculate the two important spectral densities:

$$S_{\hat{X}^\theta_{\rm det}\hat{X}^\theta_{\rm det}}(\omega) = \eta\left[|\chi^\theta_\Delta(\omega)|^2 S_{\Delta\Delta}(\omega) + |\chi^\theta_{P_{\rm in}}(\omega)|^2 2\Gamma S_{\hat{P}_{\rm in}\hat{P}_{\rm in}}(\omega) + \sum_{i=a,b,c}|\chi^\theta_{a^\dagger_{\rm in,i}}(\omega)|^2\right] + \frac{1-\eta}{2}, \quad \text{(D1)}$$

$$S_{\hat{Q}\hat{Q}}(\omega) = |\chi_m(\omega)|^2\left[|2g\chi^X_\Delta(\omega)|^2 S_{\Delta\Delta} + |1-2g\chi^X_{P_{\rm in}}(\omega)|^2 2\Gamma S_{\hat{P}_{\rm in}\hat{P}_{\rm in}}(\omega) + \sum_{i=a,b,c}|2g\chi^X_{a^\dagger_{\rm in,i}}(\omega)|^2\right] \quad \text{(D2)}$$

The single sided spectrum of Eq. D1 is used in the fitting of the measured homodyne spectral data, then $\int \frac{d\omega}{2\pi} S_{\hat{Q}\hat{Q}}(\omega) = \overline{n}_m + \frac{1}{2}$ is used to extract the unconditional mechanical thermal occupancy $\overline{n}_m$. A specific example is provided in Section G.

Section E: Multimode state estimation model

Here, starting from the quantum master equation, we derive the equations of quadrature evolution used for implementing the Kalman filter. the continuous position measurement of an oscillator at frequency Ω_m can be viewed as a form of heterodyne measurement of two orthogonal mechanical quadratures of motion $\hat{X}$ and $\hat{Y}$ that rotate with frequency Ω_m. IQ demodulation can be carried out at the mechanical frequency Ω_m. This results in two independent measurement channels of two orthogonal mechanical quadratures with independent measurement noises.

We work in a parameter regime where the measurement rate is significantly smaller than the frequency of the mechanical mode, such that we can perform IQ demodulation of the mechanical motion at Ω_m to obtain the slowly-varying X, Y quadratures. Their evolution is described by decoupled quantum master equations [4]. In the case where the measurement rate approaches the mechanical frequency, as can be in the case of resonators with a fundamental mode isolated in frequency from the higher order modes, the two equations can be coupled as was in the case in [5, 6], and measurement-induced mechanical squeezing can happen. In our case, since both quadratures are measured with identical measurement rates, only thermal coherent states are prepared through the measurement process. These states are essentially thermal states displaced from the origin of the phase space, and belong to the larger group of Gaussian states.

Since we operate in the bad-cavity limit $\Omega_m \ll \kappa$, the cavity dynamics is simplified in our modeling. Before the IQ demodulation, the normalized photocurrent signal is described by:

$$i(t) = dW(t) + \sum_i \sqrt{8\Gamma^i_{\rm meas}}\langle\hat{Q}_i\rangle(t),$$

where multiple mechanical modes $\hat{Q}_i$ oscillating at Ω_i are probed with measurement rates $\Gamma^i_{\rm meas}$, and the Wiener increment (or innovation) $dW(t) = \xi(t)dt$ is defined in terms of an ideal unit Gaussian white noise process $\langle\xi(t)\xi(t')\rangle = \delta(t-t')$. Since all oscillation frequencies are close to Ω_m, we decompose the mechanical motion into two slowly varying quadrature observables as $\hat{Q}_i = \cos(\Omega_m t)\hat{X}_i + \sin(\Omega_m t)\hat{Y}_i$, and perform IQ demodulation at frequency Ω_m, which leads to the signal in a vector form:

$$\mathbf{i}(t)dt = d\mathbf{W}(t) + \sum_i \sqrt{4\Gamma^i_{\rm meas}}\langle\hat{\mathbf{r}}_i\rangle(t)dt \quad \text{(E1)}$$

where $\mathbf{i} = \begin{bmatrix} i_X \\ i_Y \end{bmatrix}$, $\hat{\mathbf{r}}_i = \begin{bmatrix} \hat{X}_i \\ \hat{Y}_i \end{bmatrix}$ and $d\mathbf{W} = \begin{bmatrix} dW_X \\ dW_Y \end{bmatrix}$.

Since the measurement is purely linear, the system stays in Gaussian state-space [7] and the dynamics are completely captured by the expectation values of the quadratures $\langle X_i\rangle$, $\langle Y_i\rangle$, and their covariance matrix $\mathbf{C}$. To derive the time evolutions of these quantities, we start from the quantum master equation for the system density matrix $\hat{\rho}$ [4]:

$$d\hat{\rho} = -\frac{i}{\hbar}\left[\hat{H}_0, \hat{\rho}\right] + \mathcal{L}_{\rm env}\hat{\rho}dt + \sum_j \mathcal{D}[\hat{c}_j]\hat{\rho}dt + \sqrt{\eta_d}\sum_j \mathcal{H}[\hat{c}_j]\hat{\rho}dW_j,$$

where the index j is summed over X and Y measurement channels, and η_d is the total detection efficiency. The measurement observables are $\hat{\mathbf{c}}(t) = \sum_i \sqrt{\Gamma^i_{\rm qba}}\hat{\mathbf{r}}_i$. The system Hamiltonian $\hat{H}_0 = \sum_i \hbar(\Omega_i - \Omega_m)b^\dagger_i b_i$ describes the oscillator unitary dynamics in the Ω_m rotating frame. The environmental coupling term $\mathcal{L}_{\rm env}\hat{\rho}$ describes the bath induced decoherence dynamics, and with the Markov approximation in the rotating frame we have:

$$\mathcal{L}_{\rm env}\hat{\rho} = \sum_i \Gamma^i_m(\overline{n}_i + 1)\mathcal{D}[\hat{b}_i]\hat{\rho} + \Gamma^i_m\overline{n}_i\mathcal{D}[\hat{b}^\dagger_i]\hat{\rho}.$$

From here, we derive the time evolution of the quadrature expectation values as:

$$d\langle\hat{\mathbf{r}}_i\rangle = \mathbf{A}_i\langle\hat{\mathbf{r}}_i\rangle dt + 2\mathbf{B}_i d\mathbf{W}(t), \quad \text{(E2)}$$

where

$$\mathbf{A}_i = \begin{bmatrix} -\Gamma^i_m/2 & \Omega_i - \Omega_m \\ \Omega_m - \Omega_i & -\Gamma^i_m/2 \end{bmatrix}$$

and

$$\mathbf{B}_i = \begin{bmatrix} \sum_j \sqrt{\Gamma^j_{\rm meas}}C_{\hat{X}_i\hat{X}_j} & \sum_j \sqrt{\Gamma^j_{\rm meas}}C_{\hat{X}_i\hat{Y}_j} \\ \sum_j \sqrt{\Gamma^j_{\rm meas}}C_{\hat{Y}_i\hat{X}_j} & \sum_j \sqrt{\Gamma^j_{\rm meas}}C_{\hat{Y}_i\hat{Y}_j} \end{bmatrix}.$$

The covariance matrix elements $C_{\hat{M}\hat{N}} = \langle\hat{M}\hat{N} + \hat{N}\hat{M}\rangle/2 - \langle\hat{M}\rangle\langle\hat{N}\rangle$ evolve as:

$$\dot{C}_{\hat{M}_i\hat{N}_j} = -\frac{\Gamma_m^i + \Gamma_m^j}{2}\dot{C}_{\hat{M}_i\hat{N}_j} + \delta_{\hat{M}_i,\hat{N}_j}\Gamma_{\text{th}}^i + \delta_{M,N}\sqrt{\Gamma_{\text{qba}}^i\Gamma_{\text{qba}}^j}$$
$$+(-1)^{\delta_{M,Y}}(\Omega_i - \Omega_m)C_{\hat{\mathcal{M}}_i\hat{N}_j} + (-1)^{\delta_{N,Y}}(\Omega_j - \Omega_m)C_{\hat{M}_i\hat{\mathcal{N}}_j}$$
$$-4\left(\sum_k \sqrt{\Gamma_{\text{meas}}^k}C_{\hat{M}_i\hat{X}_k}\right)\left(\sum_l \sqrt{\Gamma_{\text{meas}}^l}C_{\hat{N}_j\hat{X}_l}\right)$$
$$-4\left(\sum_k \sqrt{\Gamma_{\text{meas}}^k}C_{\hat{M}_i\hat{Y}_k}\right)\left(\sum_l \sqrt{\Gamma_{\text{meas}}^l}C_{\hat{N}_j\hat{Y}_l}\right), \tag{E3}$$

where $\hat{\mathcal{M}}$ and $\hat{\mathcal{N}}$ are the canonical conjugate observables of $\hat{M}$ and $\hat{N}$.

Eqs.(E1,E2,E3) form a closed set of update equations given the measurement record $\mathbf{i}(t)$, and allow quadrature estimations of an arbitrary number of modes and their correlations. The thermal occupancy $\overline{n}_{\text{cond}}^i$ of a specific mechanical mode is determined by the quadrature phase space variances $V_{\hat{X}_i} = C_{\hat{X}_i\hat{X}_i}$, $V_{\hat{Y}_i} = C_{\hat{Y}_i\hat{Y}_i}$, which are both equal to $\overline{n}_{\text{cond},i} + 1/2$ due to the symmetric measurement rates of the X, Y channels.

In the case of the evolution of a single mechanical mode, the quadrature variance approaches a steady state described by an analytical solution,

$$V_{X,Y} = \frac{-\Gamma_m' + \sqrt{\Gamma_m'^2 + 16\Gamma_{\text{meas}}(\Gamma_{\text{th}} + \Gamma_{\text{qba}})}}{8\Gamma_{\text{meas}}}, \tag{E4}$$

where $\Gamma_m' = \Gamma_m + \Gamma_{\text{opt}}$ takes into account the optical damping rate Γ_{opt}. For a system consisting of multiple mechanical modes, that are not sufficiently separated in frequency ($|\Omega_i - \Omega_j|$ not significantly faster than any other rates in the system), cross correlations between different mechanical modes emerge due to common measurement imprecision noise and common quantum backaction force. This generally leads to higher quadrature variance due to the effectively reduced measurement efficiency of individual modes.

To decouple the mechanical oscillators that are interacting due to the spectral overlap and the measurement process, we define a new set of collective motional modes through a symplectic (canonical) transformation $\mathbf{U}$ of quadrature basis that diagonalizes the steady state covariance matrix $\mathbf{U}^\dagger\mathbf{C}\mathbf{U} = \mathbf{V}$ [8]. Since the covariance matrix is real and symmetric, the elements of $\mathbf{U}$ are always real, which is required for real observables. The transformation can be understood as a normal mode decomposition of the collective Gaussian state that preserves the commutation relations, as opposed to conventional diagonalization using unitary matrices. This is represented by the requirement of the symplectic transformation $\mathbf{U}\mathbf{\Omega}\mathbf{U}^\dagger = \mathbf{\Omega}$ where $\mathbf{\Omega} = \begin{bmatrix} 0 & \mathbf{I}_N \\ -\mathbf{I}_N & 0 \end{bmatrix}$ is the N-mode symplectic form.

For the multimode estimation experiment, we extract the required system parameters of the nearest 10 membrane mechanical modes around Ω_m by fitting the measured spectral noise density, and feed the time-series signal $\mathbf{i}(t)$ to the closed update equations to retrieve all the 20 quadrature expectations and 210 independent covariance matrix elements at different times. The signal is normalized such that the Wiener increment gives the correct noise level in frequency domain, and the filtered unconditional defect mode signal has the correct laser-cooled thermal occupancy. The prediction-retrodiction method is used to reconstruct the covariance matrix $\mathbf{C}$, which is then used to compute the symplectic transformation needed to define the new collective mode bases. We derived the retrodiction update equations and found that they are identical to the prediction update equations [9], except with negative mechanical frequencies. As a result, we have the following relations between covariance matrix elements estimated by prediction and retrodiction (respectively identified by the superscripts p and r):

$$C^p_{\hat{X}_i\hat{X}_j} = C^r_{\hat{X}_i\hat{X}_j}$$
$$C^p_{\hat{Y}_i\hat{Y}_j} = C^r_{\hat{Y}_i\hat{Y}_j}$$
$$C^p_{\hat{X}_i\hat{Y}_j} = -C^r_{\hat{X}_i\hat{Y}_j}.$$

Section F: Numerical implementation of multimode state estimation

We record the voltage output from the photodetector using an UHFLI lock-in amplifier (Zurich Instruments), for a total duration of 2 s, digitizing the signal at 14 MHz sampling rate, and we store the data digitally for post-processing. The noise power spectrum density of the digitized signal is compared to the one simultaneously measured on a real-time spectrum analyzer, to rule out SNR degradation from the digitization noise. Two 7th-order Butterworth bandpass filters with passband 1.05-1.22 MHz are applied in order to reduce the influence of the mechanical modes outside the bandgap, and around 50 notch filters are applied to these mechanical modes until no mechanical peak is above the shot noise level. After the filtering, only the 10 mechanical modes around the defect mode frequency Ω_m need to be kept for the multimode state estimation study.

As discussed in the previous section, we demodulate the signal at Ω_m, and implement the discretized version of the update equation Eq. E2,

$$\Delta\langle\hat{\mathbf{r}}_i\rangle = \mathbf{A}_i'\langle\hat{\mathbf{r}}_i\rangle\Delta t + 2\mathbf{B}_i\Delta\mathbf{W}(t) \tag{F1}$$

where $\mathbf{A}_i' = \begin{bmatrix} -\Gamma_m'^i/2 & \Omega_i' - \Omega_m \\ \Omega_m - \Omega_i' & -\Gamma_m'^i/2 \end{bmatrix}$ contains modified mechanical parameters:

$$\Gamma_m'^i = \Gamma_m^i + 2\text{Re}\left[-\frac{1 - \cos((\Omega_i - \Omega_m)\Delta t)}{\Delta t}\right]$$
$$\Omega_i' = \Omega_i - \text{Im}\left[i(\Omega_i - \Omega_m) - \frac{e^{i(\Omega_i - \Omega_m)\Delta t} - 1}{\Delta t}\right]$$

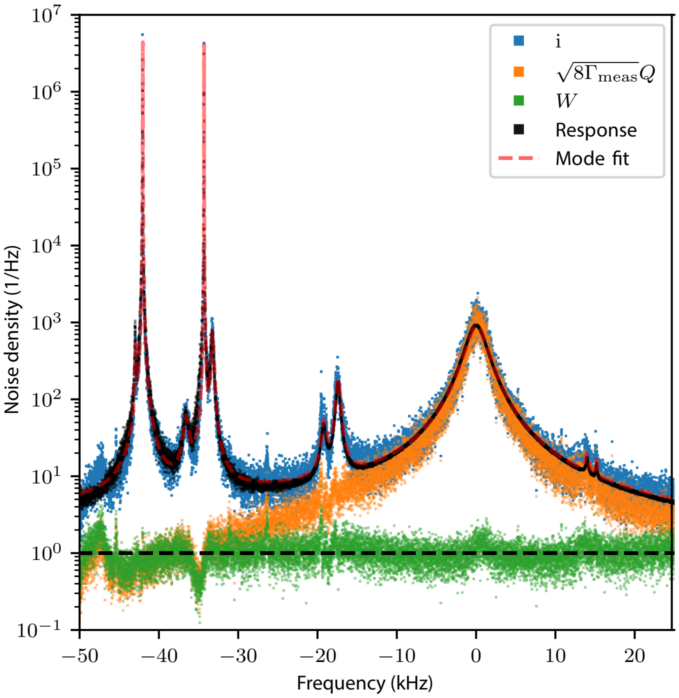

FIG. F.1. Multimode state estimation spectral response. The noise density of the photocurrent signal $i(t)$ is shown in blue, together with the fit (dashed red), employed to extract the system parameters for the 10 mechanical modes in this frequency band. Those parameters are used to construct the multimode state estimation filter, from which the mechanical mode signals (defect mode shown in orange) and the measurement imprecision (green) are separated. The white noise filter frequency response (gray) is numerically retrieved, and is identical to that of the spectral fit, indicating that the multimode state estimation filter is correctly implemented.

to compensate for the influence of discretization on the state estimation performance compared to an ideal continuous one. The matrix $\mathbf{B}_i$ evolution can be computed independently from the sampled time-domain data. Therefore, we calculate the evolution following Eq. E3, with an update rate of 140 MHz to mitigate the discretization effect, which is then used for the update equation Eq. F1 at the sampling rate of 14 MHz. The correct implementation of the state estimation can be checked from the Kalman filter response numerically calculated from the estimated $i(t)$, $Q_i(t)$ and $W(t)$, and is shown in Fig. F.1. Another important feature that validates our modelling is the white noise property of our numerically retrieved $W(t)$, evident from its spectral property.

To experimentally reconstruct the covariance matrix from the estimated quadrature data, for each time trace slice we calculate the difference between prediction and retrodiction results $\langle\hat{\mathbf{r}}\rangle_r - \langle\hat{\mathbf{r}}\rangle_p$, and calculate the covariance matrix via

$$\mathbf{C} = \frac{1}{2}\langle\!\langle (\langle\hat{\mathbf{r}}\rangle_r - \langle\hat{\mathbf{r}}\rangle_p)\cdot(\langle\hat{\mathbf{r}}\rangle_r - \langle\hat{\mathbf{r}}\rangle_p)^\top \rangle\!\rangle$$

where $\langle\!\langle\cdots\rangle\!\rangle$ is the statistical average over all the time trace slices, and $\hat{\mathbf{r}} = \left[\cdots\hat{X}_i, \hat{Y}_i\cdots\right]^\top$. The covariance matrix is then diagonalized to retrieve the symplectic transformation for the collective modes, and the thermal occupations of these modes. We find that in the new quadrature basis based on the diagonalized covariance matrix, the modified defect mode is only weakly modified, and the thermal occupancy can reach that of the single-mode optimal limit $\overline{n}_{\text{cond,single}} = 0.88$. The transformation coefficents for the defect mode $\hat{X}$ is shown in the following table. Note that the contributions from $\hat{Y}_i$ are attributed to statistical uncertainties.

$\Omega_i - \Omega_m$ (kHz)	Coefficients $\hat{X}$	Coefficients $\hat{Y}$
0	0.999	5.05×10^{-7}
15.1	1.53×10^{-5}	-2.11×10^{-6}
13.9	-2.54×10^{-7}	-2.33×10^{-6}
-17.4	2.16×10^{-4}	5.06×10^{-6}
-19.3	1.23×10^{-4}	2.11×10^{-6}
-33.3	3.04×10^{-4}	3.14×10^{-6}
-34.4	2.96×10^{-2}	5.27×10^{-4}
-36.6	1.15×10^{-4}	-8.88×10^{-6}
-42.1	4.18×10^{-2}	-2.13×10^{-4}
-43.0	1.50×10^{-4}	3.14×10^{-6}

Section G: Sideband cooling limit

In our system, due to the reflectivity wavelength dependence of the dielectric coatings of the cavity, we can choose the optical linewidth by tuning the laser to a different optical wavelength. We test the sideband cooling phonon occupancy limit of our system with an optical mode at 862.2 nm with optical linewidth $\kappa/2\pi$ = 13.5 MHz, the lowest among all the optical modes we characterized. The idealized theoretical cooling limit is:

$$\overline{n}_{\text{ideal}} = \frac{(\Omega_m + \bar{\Delta})^2 + (\kappa/2)^2}{-4\bar{\Delta}\Omega_m} = 2.9,$$

evaluated with the laser pump applied at the magic detuning. We pump the cavity with 2 mW of input power and measure the output spectrum of the cavity (see Fig. G.1(a,c)). Using Eq. D1 to fit the measured spectrum, we can reliably extract all the system parameters ($\mathcal{C}_q$, η, θ), and also the noise from the nearby spurious mechanical modes $\bar{S}_{\Delta\Delta}(\omega)$. Using the extracted quantities, we can reconstruct the mechanical position noise density $\bar{S}_{QQ}(\omega)$ using Eq. D2 (see Fig. G.1(b,d)).

To estimate the lowest achievable phonon occupancy, we consider two mechanical mode bases. In the original mode basis, with the full model described by Eq. D1 and Eq. D2, the sideband cooling mediates a coupling between mechanical modes. As a result, the correlation between the defect mode and the spurious modes is non-negligible, and leads to a phonon occupancy of the defect mode greater than the theoretical ideal. Under our experimental conditions, the best phonon occupancy with

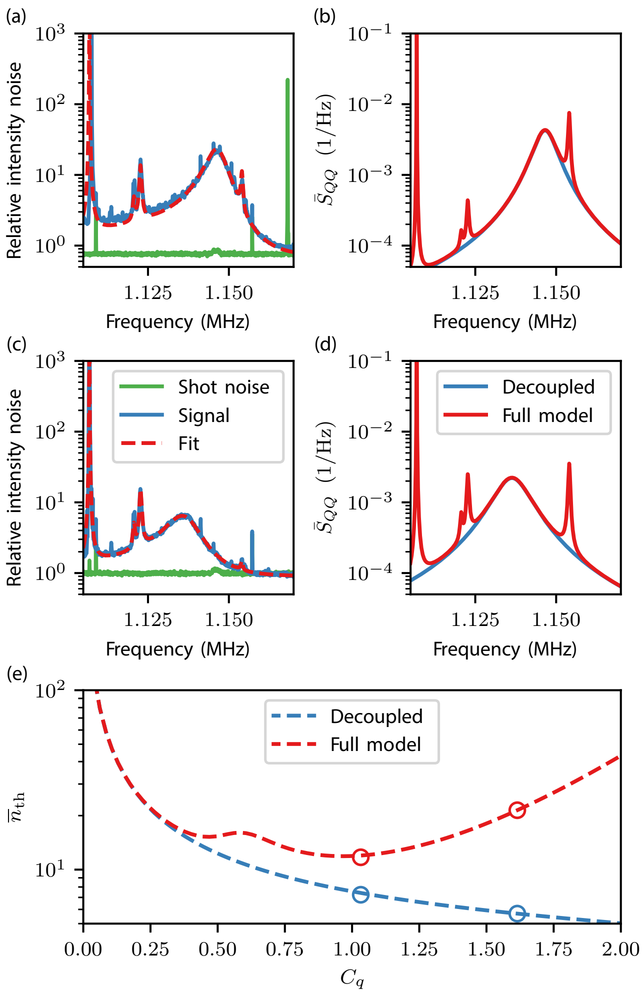

FIG. G.1. Sideband cooling limit study using the 862.2 nm optical mode. **(a)** Measured mechanical spectrum at $\mathcal{C}_q$ = 1.0, showing spectral overlap between membrane modes. **(b)** From the fit, the position noise density of the defect mode is reconstructed, and shows correlations to the nearby auxiliary modes. **(c,d)** Same measurement and analysis but for $\mathcal{C}_q$ = 1.6. **(e)** Calibrated sideband cooling phonon occupancy at different $\mathcal{C}_q$.

this optical mode was $\overline{n}_{\rm eff} = 11.7$ at relatively low optical powers ($\mathcal{C}_q = 1.0$, see Fig. G.1(e)).

Next, we analyze the system in the framework of multimode optomechanics [10] where the cavity-induced coupling between mechanical modes results in mode hybridization and a new set of decoupled normal mode basis. In the decoupled basis, where the cooling-induced correlations between the mechanical modes are nulled, the decoupled defect mode achieves a phonon occupancy $\overline{n}_{\rm eff} = 5.7$ at $\mathcal{C}_q = 1.6$. This is a result of the small number of correlation quanta (~ 10) compared to the large phonon occupancy ($\gg 10$) of the spurious modes in the original basis. Such analysis is similar to those presented in the previous cooling experiments [11, 12], where spurious mechanical modes near the target mode couple to the optomechanical cavity. Essentially, by fitting a Lorentzian to the defect mode and ignoring nearby spurious modes, one attains the same phonon occupancy as the decoupled mode. The obtained phonon occupancy is also identical to that which would be retrieved by an independent sideband asymmetry measurement [5]. We stress that the phonon occupancy evaluated this way is only associated with the decoupled mode, which is defined in the presence of the cooling pump. In applications that require removal of the cooling pump, the mechanical modes decouple and the effective phonon occupancy goes back to that of the full model. At the experimental condition of $\mathcal{C}_q = 1.6$, the full model would yield an increased phonon occupancy of $\overline{n}_{\rm eff} = 21.4$ (see Fig. G.1(e)), still among the lowest phonon occupancy achieved in nanomechanical devices. Generally, the cooling-induced multimode hybridization results in higher total phonon occupancy compared to the idealized case, but in some special cases the decoupled modes can have lower occupancies.

We further stress that the correlated quanta evaluated from the full model do not impose any limit on measurement-based feedback schemes. In the presence of sideband cooling, the cooling-induced correlations to the spurious modes can be completely cancelled in a measurement-based feedback scheme. When the defect mode experiences both a thermal force $\sqrt{2\Gamma_m}\hat{P}_{\rm in}$ and a feedback force $\hat{F}_{\rm fb}$, where the feedback force is determined by the measurement record $\hat{F}_{\rm fb}(\omega) = H(\omega)\hat{X}^{\theta}_{\rm det}(\omega)$, a linear filter $H_0(\omega) = -2g/\sqrt{\eta\kappa_a}$ completely cancels the cooling induced correlations. This can be derived by the following expression (Fourier frequency dependence hidden) of spurious-mode-induced heating of the target mode

$$S_{QQ} = |\chi_m|^2 |\chi_F \chi_H \chi^{\theta}_{\Delta} - 2g\chi^X_{\Delta}|^2 S_{\Delta\Delta} + \cdots$$

$$\chi_F = 1 - 2g\chi^X_{P_{\rm in}}$$

$$\chi_H = \frac{H}{\sqrt{\eta}^{-1} - \chi^{\theta}_{P_{\rm in}} H}$$

Therefore, for any measurement-based control scheme that requires a filter $H_1(\omega)$, the constructed filter $H(\omega) = H_0(\omega) + H_1(\omega)$ achieves the desired state with no correlation to spurious modes.

Section H: Mechanical simulations of density-modulated PnC membranes

The quality factor of the vibrational mode of a strained resonator subject to dissipation dilution is given by $Q = D_Q \times Q_{\rm int}$, where $Q_{\rm int}$ is the inverse of the resonator material's loss tangent, and D_Q is the dilution factor [13]. The dilution factor depends on the mode deformation pattern, $\vec{u} = \vec{u}(x,y)$, and on the resonator geometry. It can be estimated analytically or numerically. For high aspect ratio resonators (large lateral extent, small thickness) and for flexural vibrations, the dilution factor can be extremely large (up to $10^5 - 10^6$ [14, 15]) and the quality factor

is correspondingly enhanced beyond the material inverse loss tangent. To compute dilution factors, we carry out pre-stressed eigenfrequency analyses in COMSOL Multiphysics, using the "Shell" interface. This finite elements study is particularly suitable for the simulation of high aspect ratio nanomechanical objects.

The dilution factor depends strongly on the mode curvature close to the clamping points [16]. For this reason, we refine the mesh for the finite element model close to the membrane clamped boundaries (outer edges), using a typical element size around $\lambda L/30$. Here, L is the largest lateral dimension of the membrane, $\lambda = \sqrt{Eh^2/(12\sigma L^2)} \ll 1$ is the strain parameter, E is the membrane Young's modulus, σ is the material stress, and h is the membrane thickness. Fixed boundary conditions are imposed at the membrane clamping points, i.e. $\vec{u} = \partial\vec{u}/\partial\vec{n} = 0$ (where $\partial\vec{u}/\partial\vec{n}$ is the displacement field derivative normal to the boundary).

The dilution factors are calculated with the ratio of kinetic and linear elastic energies [13]. For out-of-plane bending modes:

$$D_Q = \frac{12\rho\Omega^2}{Eh^2}\frac{\left(1-\nu^2\right)\int w^2\ dx\,dy}{\int\left(\partial^2 w/\partial x^2 + \partial^2 w/\partial y^2\right)^2 + 2\left(1-\nu\right)\left(\left(\partial^2 w/\partial x\partial y\right)^2 - \partial^2 w/\partial x^2\cdot\partial^2 w/\partial y^2\right)dxdy}, \tag{H1}$$

where w is the u component in the out-of-plane direction, Ω is the mechanical frequency, ρ is the mass density of the membrane material and ν is Poisson's ratio. A PnC nanomechanical structure can enhance the D_Q of some bending resonances, by localizing the displacement to a defect in the spatial-periodic modulation of the speed of sound and suppressing its curvature close to the clamping boundaries. This idea, introduced by Tsaturyan et al. in 2016 [17], was the first incarnation of the "soft clamping" effect.

In our case, the PnC is realized with a modulation of the resonator's effective mass density, described by $\rho_{\text{eff}}(x,y) = g(x,y)\rho$ [18]. In practice, the density modulation is implemented by patterning nanopillars over the nanomechanical membrane, with a thickness much larger than that of the membrane. Pillars are confined to circular regions of diameter commensurate with the membrane acoustic wavelength (see Fig. 1(f)), and are arranged in a triangular pattern. The nanopillars locally mass-load the membrane to realize a density modulation, but they also introduce additional mechanical dissipation. When a bending wave impinges on the pillar, it induces deformation carrying linear strain energy without a significant geometrically-nonlinear contribution [13]. The pillars will thus reduce the dissipation dilution of the Si_3N_4 membrane, by an amount that depends sensitively on the pillars' geometrical dimensions and on the elastic frequency of the flexural mode. The lower the elastic frequency and the smaller the pillars, the lower the susceptibility of the pillar displacement, and the lower the added dissipation induced by the individual pillars will be. The pillar damping contribution can be dominant, or sufficiently low that it is negligible compared to the finite dissipation dilution of the soft clamped mode, depending on the oscillation frequency and on the pillars' geometry [18].

We investigated this damping contribution with 3D finite element simulations of a single nanopillar unit cell. From now on, we assume that the pillars are arranged in a triangular lattice with a lattice constant a_{pil} (separation between the nearest neighbours), and we identify the pillar diameter and height (thickness) with d_{pil}, h_{pil}. The effective density in the circular regions patterned with the nanopillars is then:

$$\rho_{\text{eff}} = \rho\left[1 + \frac{\pi}{2\sqrt{3}}\frac{\rho_{\text{pil}}h_{\text{pil}}}{\rho h}\left(\frac{d_{\text{pil}}}{a_{\text{pil}}}\right)^2\right], \tag{H2}$$

where h and ρ are the thickness and density of the membrane film (Si_3N_4). The periodicity of the pillar array is embedded in the simulation by restricting the domain to an hexagon-shaped unit cell (see Fig. H.1(a)) with Floquet boundary conditions $[\vec{u}(\vec{x}+\vec{R}) = \vec{u}(\vec{x})e^{-i\vec{k}\cdot\vec{R}}]$ on opposing sides of the hexagon. The magnitude of the elastic wavevector that defines the boundary conditions, k, is chosen in order to produce a flexural eigenmode at the mechanical frequency of interest ($ka_{\text{pil}} \ll 1$):

$$k \approx \Omega\sqrt{\frac{\rho_{\text{eff}}}{\sigma}}, \tag{H3}$$

where σ is the membrane deposition stress and Ω is the mechanical frequency in angular units. The model is then solved for its first eigenmode, which represents the pillar displacement upon the arrival of the flexural wave. The dissipation dilution of an infinite pillar lattice is evaluated using Equation H1, with w corresponding now to the z component of the displacement field of the nanopillar. The results of the computation of the infinitely-extended pillar lattice's quality factor are shown in Fig. H.1(b), for a fixed pillar thickness of $h_{\text{pil}} = 450\,\text{nm}$ and separation $a_{\text{pil}} = 2\,\mu\text{m}$, and variable diameters and mechanical frequencies. The method was also benchmarked by verifying that when the pillar thickness is set to 0, the extracted dilution factor is given by the "clampless" ex-

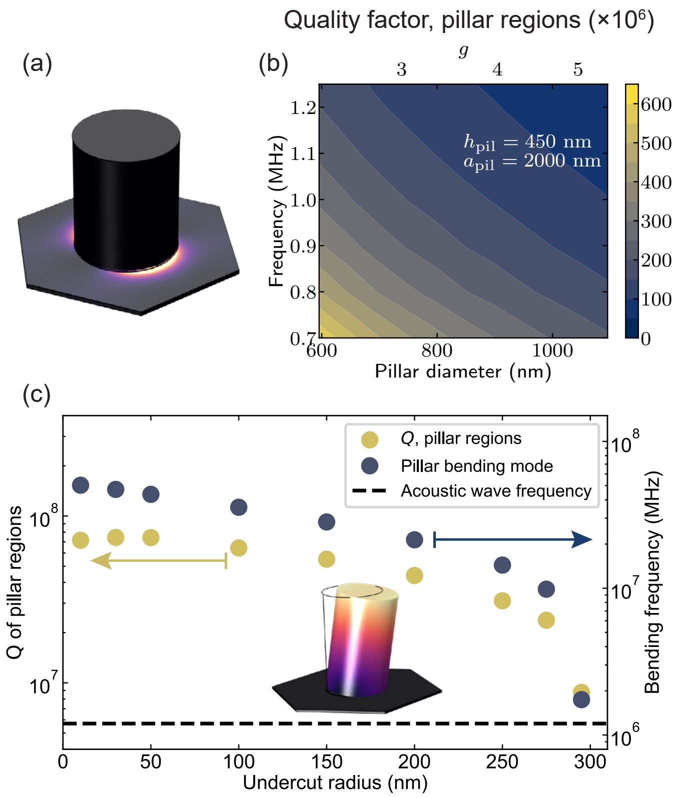

FIG. H.1. Losses induced by bending of nanopillars used to modulate $\rho_{\rm eff}$. **(a)**, Visualization of the linear elastic energy density, which is associated with mechanical dissipation, close to the nanopillar. The largest contribution to dissipation is seen to occur close to the nanopillar base. **(b)**, Q of a uniform pillar array with infinite extent as a function of the frequency of the acoustic flexural wave and of the pillar diameter. The pillar separation $a_{\rm pil}$ and the pillar thickness $h_{\rm pil}$ are kept constant to the specified values. The horizontal axis is expressed in terms of the pillar diameter and of the resulting density modulation $g = \rho_{\rm eff}/\rho$. **(c)**, Effect of an undercut at the pillar base on the pillar Q. For this simulation, the pillar has a diameter of 600 nm and a thickness of 1000 nm, and the separation between nearest-neighbours pillars is fixed to 1.5 µm. The undercut layer is hardly visible in the illustration, as it is only 6 nm-thick. The pillar array's Q (ochre dots) is seen to drop sharply when the first flexural resonance frequency (blue dots, the inset illustrates the displacement field) of the nanopillar approaches the acoustic excitation frequency, marked by a horizontal dashed line.

pression [13]:

$$D_Q \approx \frac{12(1-\nu^2)\sigma^2}{E\rho_{\rm min}h^2\Omega^2}, \tag{H4}$$

Note that the aSi loss angle is an unknown parameter in the FEM simulations, that we arbitrarily set by choosing $Q_{\rm int,aSi} = 10000$. The results in Fig. H.1(b) do not depend sensitively on $Q_{\rm int,aSi}$; nevertheless one should treat them as a rough estimation of the pillar damping contribution.

Another pillar geometry that is experimentally relevant is the case of a cylindrical pillar with a thin undercut layer formed at the base. As described in the next section, this is a possible outcome of the nanopillar microfabrication, in case the pillar is grown on top of an etch-stop layer with a different chemical identity. In this scenario, the frequency of the first bending mode of the nanopillars is decreased, as shown by the blue dots in Fig. H.1(c), and the pillar motion can start to hybridize with the soft clamped membrane mode. This induces a large reduction in the overall quality factor when the undercut covers a significant portion of the pillar base (see the yellow dots in Fig. H.1(c)). Note that the undercut is not visible in the figure inset, as it is carved in a layer of only 6 nm at the pillar base, which is similar to the fabricated pillar geometrical parameters.

In real density-modulated PnC devices, the nanopillar lattice does not cover the full membrane but is limited to the periodic circular regions of high density (see Fig. 1(e)). To compute the actual damping contribution due to the pillars, it is then necessary to weigh the quality factor calculated as before with a *participation ratio* of the linear elastic energy developed in the high-$\rho_{\rm eff}$ regions. The overall dissipation limit is then estimated using the following formula:

$$\begin{aligned} Q_{\rm pillar\ bend} &= Q_{\rm single\ pillar}/p_{\rm hd} \\ p_{\rm hd} &= \frac{\int_{\rm hd} dS\, \left(\left(\frac{\partial^2 w}{\partial x^2}+\frac{\partial^2 w}{\partial y^2}\right)^2 + 2(1-\nu)\left(\left(\frac{\partial^2 w}{\partial x\partial y}\right)^2 - \frac{\partial^2 w}{\partial x^2}\frac{\partial^2 w}{\partial y^2}\right)\right)}{\int dS\, \left(\left(\frac{\partial^2 w}{\partial x^2}+\frac{\partial^2 w}{\partial y^2}\right)^2 + 2(1-\nu)\left(\left(\frac{\partial^2 w}{\partial x\partial y}\right)^2 - \frac{\partial^2 w}{\partial x^2}\frac{\partial^2 w}{\partial y^2}\right)\right)}, \end{aligned} \tag{H5}$$

where the integral at the numerator of the participation ratio $p_{\rm hd}$ is computed only in the high-density regions, while the one at the denominator is extended to the whole membrane surface. The energy participation ratio de-

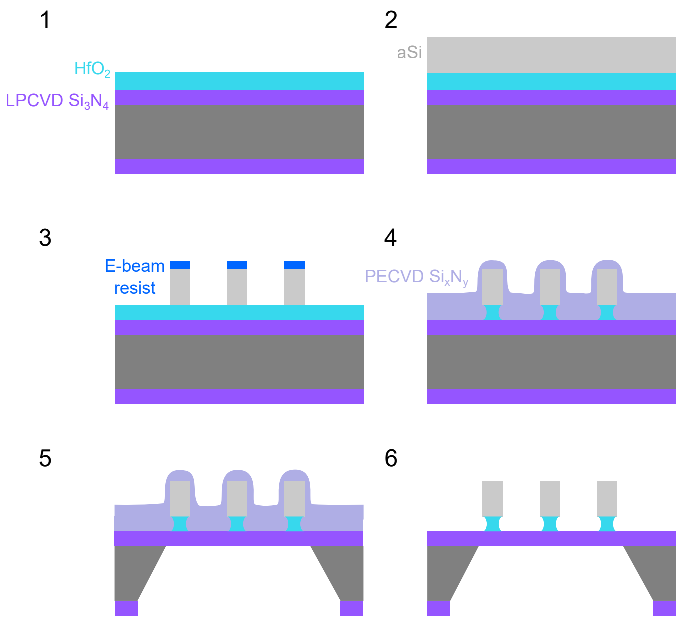

FIG. I.1. Simplified fabrication process for density-modulated membrane resonators. Layer thicknesses and lateral dimensions are not to scale. 1 – Etch stop layer growth. 2 – aSi pillar layer growth. 3 – Electron beam lithography and pillar pattern transfer with RIE. 4 – Growth of PECVD nitride encapsulation layer. 5 – Chip separation and KOH membrane release. 6 – Removal of the encapsulation layer in buffered HF.

pends sensitively on the PnC defect geometry.

For the PnC membrane device employed in these experiments, the macroscopic estimate of the quality factor, neglecting pillar dissipation, is $D_Q \times Q_{\mathrm{int}} \approx 6 \cdot 10^8$, slightly lower than the design value due to a low density modulation, $g_{\mathrm{max}} \approx 2.9$, an accidental result of fabrication drifts. The dissipation contribution due to the nanopillars turned out to be comparable, for the measured pillar dimensions: $Q_{\mathrm{pillar\ bend}} \approx 6 \cdot 10^8$. Overall, the two dissipation contributions combine to give an estimate for the overall intrinsic loss-dominated quality factor $Q \approx 3 \cdot 10^8$, slightly higher than what was measured with the membrane device. We also cannot exclude a small contribution to the observed dissipation due to collisions with the residual gas molecules in the vacuum chamber where the MIM cavity is located [19].

Section I: Microfabrication of density-modulated PnC membranes

We implement density-modulated PnC membranes by fabricating amorphous silicon (aSi) nanopillars on a high aspect ratio Si_3N_4 membrane. This method has some advantages over the original density-modulated membrane samples [18], where the pillars are made of plasma-enhanced chemical vapor deposition (PECVD) silicon nitride. For example, the dimensional accuracy of the fabricated nanopillars is improved. We also found that density-modulated membranes with PECVD nitride pillars caused a strong optical bistability of the MIM cavity [20], probably due to excess optical absorption induced in the Si_3N_4 layer during our particular PECVD process. This experimental obstacle seemingly disappeared, when we adopted new membrane samples with aSi pillars. However, the process becomes a little more laborious, due to the need of protecting the pillars during the membrane undercut. The aSi pillars and the substrate have a very similar chemical composition, and they will be dissolved during the undercut process, if exposed to the etchant (KOH). We devised a PECVD Si_xN_y encapsulation layer for the protection of aSi pillars, that can be removed selectively with respect to the pillars and the membrane as the last step of microfabrication.

In our PnC membranes, we fabricated pillars with diameters between d_{pil} = 300 to 800 nm and nearest-neighbour distances between a_{pil} = 1.0 to 2.0 µm. We chose to use electron beam lithography to pattern the pillars, for the highest versatility of prototyping. Dry etching transfers the mask pattern to the underlying film, while maintaining smooth and vertical sidewalls and keeping the pattern dimensions faithful to the original design. However, there are few dry etching recipes with a good selectivity between Si_3N_4 and Si; as soon as the ~ 20 nm Si_3N_4 membrane is uncovered, it would rapidly get consumed by the etching process. One solution is to stop the dry etching step just short of uncovering the Si_3N_4 layer, and finishing with a high-selectivity step such as wet etching [18]. Unfortunately, wet etching is typically isotropic, and would shrink the pillar dimensions from the design values, bringing forth issues in the reproducibility and control of the effective density and pillar damping. We decided to instead employ an etch-stop layer, much more resistant to dry etching than Si_3N_4, on top of the membrane film, that allows for adequate overetching and process tolerances. Such an etch-stop material is not difficult to find: for SF_6-based processes, oxides such as SiO_2, Al_2O_3 or HfO_2 (hafnium oxide) have been tested and found to provide a suitable selectivity. However, another important requirement is that no significant undercut in the etch-stop layer is created in subsequent steps of microfabrication. Undercut at the pillar base can reduce the frequency of the pillar bending resonances dramatically, thus increasing the pillar dissipation at MHz frequencies. HfO_2 proved to satisfy this requirement, as a very thin layer with thickness < 5 nm can completely block the dry etching step for a sufficient amount of time, and it is dissolved in many acid and base solutions.

The process starts with (100)-oriented silicon wafers on which a ~ 20-nm layer of stoichiometric, high stress Si_3N_4 has been grown via low-pressure chemical vapor deposition. After cleaning and dehydrating the wafer with an O_2 plasma, we proceed to grow with atomic layer deposition (ALD) a ~6-nm HfO_2 etch-stop

film (step 1 in Fig. I.1). The growth takes place with a reactor temperature of 200 °C, using H_2O and Tetrakis(ethylmethylamido)hafnium (TEMAHf) as precursors. TEMAHf is pre-heated to 80 °C before starting the deposition process. We then proceed with the deposition of the aSi pillar layer. We employ a PECVD tool (Oxford PlasmaLabSystem 100), with silane (SiH_4) as the only precursor (step 2 of Fig. I.1). The chamber temperature is set to 300 °C during the deposition, and a 2% SiH_4:N_2 mixture is flowed in the chamber at 1000 sccm; plasma is generated using 30 W of RF power. The pressure in the chamber is kept around 1500 mtorr during the process. The typical pillar thickness we target is about 600 nm.

We then proceed to define the nanopillar pattern with electron beam lithography. We spin-coat flowable oxide FOx16 resist (a formulation of HSQ) at 2000 RPM, resulting in a mask layer approximately 800-nm thick. FOx is exposed with a dose of about 1400 $\mu C/cm^2$ and developed with TMAH25% (2 minutes of immersion with agitation). Before the e-beam writing, the pattern is corrected for proximity effects in electron beam exposure that would lead to nonuniformity within the pillar lattice regions. The pattern is transferred to the aSi layer with reactive ion etching (step 3 in Fig. I.1), using a recipe in which SF_6 and C_4F_8 gases flow simultaneously in the plasma chamber, where the wafer is kept at 20 °C. The etch is monitored *in situ* using a 670-nm laser beam reflected from the thin film stack: as the aSi layer is gradually thinned down, fringes are observed, due to thin film interference. The endpoint, occurring when the HfO_2 layer is exposed, is clearly visible from the sudden dip of the interference signal slope. We let the dry etching process run for about 30 s after the endpoint to ensure that the pillars are fully defined on the whole wafer, then we stop the process. Finally, we remove the FOx mask and the residual etch-stop layer by dipping the wafer in HF 1% for about 3.5 min.

After patterning the pillars, we encapsulate them in a dielectric layer to protect them during the silicon deep etching step (step 4 in Fig. I.1). We require a layer that can conformally cover the pillar topography, without defects or pinholes and with tensile deposition strain, such that it does not destructively buckle when it is suspended. Owing to previous experience in other microfabrication projects [14], we employ PECVD Si_xN_y. We first grow a thin (~ 20 nm), protective layer of Al_2O_3 with ALD, to shield the membrane layer from plasma bombardment during PECVD. Then, approximately 125 nm of Si_xN_y is grown in our Oxford PlasmalabSystem100 PECVD with 2% SiH_4:N_2 and NH_3 as the precursors. The flow rates are set to 975 and 30 sccm. The chamber pressure is 800 mtorr and the reactor temperature is kept to 300 °C during the deposition. 40 W of RF power excites the plasma during deposition, and the deposited layer has been characterized to have a tensile stress of around +300 MPa at room temperature. This PECVD layer has proved to perfectly seal the nanopillars and withstand several hours of immersion in hot KOH without significant consumption.

At this point the process proceeds analogously to that of conventional stress-modulated PnC membranes (see the Supplementary Material of [21]). A thick (~ 3 μm) layer of positive tone photoresist is spun on the frontside for protection during the backside lithography process that we perform with an MLA150 laser writer (Heidelberg Instruments), with alignment to 8 frontside markers. Membrane windows must be appropriately resized in order to account for the KOH slow-etching $\langle 111 \rangle$ planes. UV lithography is followed by Si_3N_4 dry etching with a plasma of CHF_3 and SF_6. After the resist mask and protection layer removal with N-Methyl-2-Pyrrolidone (NMP) and O_2 plasma, we deep-etch with KOH from the membrane windows while keeping the frontside protected, by installing the wafer in a watertight PEEK holder where only the backside is exposed (see [17, 21]). KOH40% at 70 °C is employed, and the etching is interrupted when about 30-40 μm of silicon remain. The wafer is then rinsed and cleaned with hot HCl of the residues formed during KOH etching. Then, the wafer is separated into individual dies before concluding the process: a protective layer of positive-tone resist is coated on the frontside before cutting the wafer with a dicing saw, and the process continues chipwise. Chips are again cleaned with NMP and O_2 plasma, and the deep-etch is concluded with a second immersion in KOH 40% at a lower temperature of 55 °C (step 5 in Fig. I.1), followed by cleaning in HCl. From the end of the KOH etching step, the composite membranes are suspended, and great care must be adopted in displacing and immersing the samples in liquid; nevertheless, the presence of a relatively thick PECVD nitride layer ensures that the survival yield is quite high after this step (> 90%). We dry the samples by moving them to an ultrapure isopropyl alcohol (IPA) bath after water rinsing. IPA has a low surface tension and a high vapour pressure, therefore it can be easily dried off the membranes after a few minutes of immersion, with the help of a (cautiously operated) N_2 gun.

Finally, the PECVD nitride and Al_2O_3 layers can be removed selectively with wet etching in buffered HF (BHF; step 6 in Fig. I.1). The etch rates of the encapsulation layers in BHF are orders of magnitude higher than the etch rate of stoichiometric Si_3N_4 and HfO_2, therefore even though the membrane backside is exposed, the membrane thinning during this step remains limited (few nanometers). Chips are loaded in a Teflon carrier where they are vertically mounted, and immersed for about 3 min 20 s in BHF 7:1. It is crucial not to overetch more than necessary to fully remove the encapsulation films: membranes become extremely fragile and the survival yield drops sharply when their thickness is reduced below ~ 15 nm. The membranes are then carefully rinsed, transferred in an ethanol bath and dried in a critical point dryer, where the liquids can be evacuated gently and with little contamination.

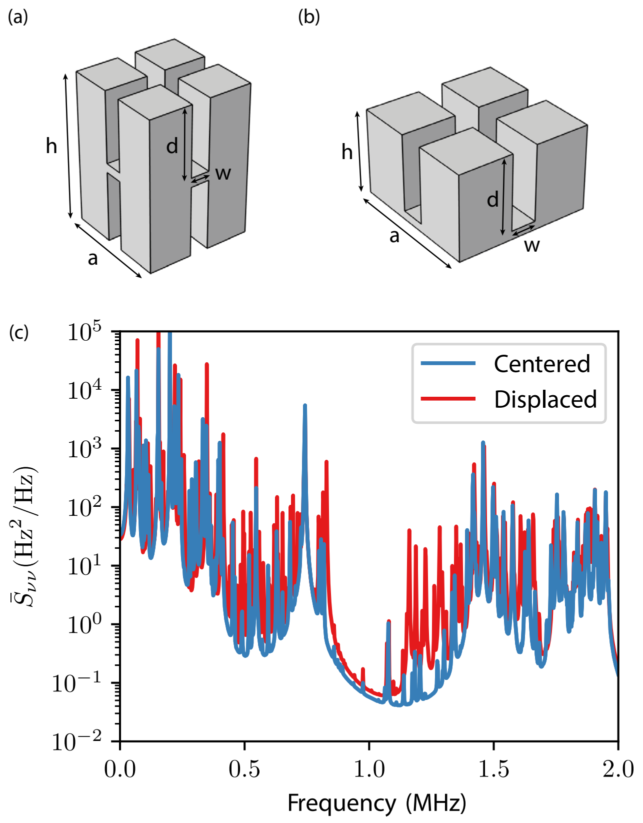

FIG. J.1. **a** Square lattice unit cell of the top mirror. The periodicity is along the horizontal plane. a=1.4mm, h=4mm, d=1.88mm, w=250µm. **b** Square lattice unit cell of the bottom mirror. The periodicity is along the horizontal plane. a=1.6mm, h=1mm, d=900µm, w=300µm. **c** Simulated frequency noise spectra of an empty cavity with PnC top and bottom mirrors. We show both the case of an optical mode perfectly centered on the bottom mirror (blue), and displaced 200 µm away from the center (red), accounting for a realistic assembly misalignment.

Section J: Fabrication and simulation of phononic crystal mirrors

Using PnC structures to reduce cavity frequency noise from thermomechanical motion of the mirrors was previously explored in Ref. [12]. In that work, the bottom mirror is fabricated by first patterning a silicon wafer with a periodic lattice of holes, and then bonding it with a Pyrex substrate on which the mirror coating is sputtered. Because of the technical difficulty in applying the same procedure for the curved top mirror, a fiber mirror is used instead, which also serves as the high transmission port of the cavity. The use of a fiber mirror resulted in excess broadband cavity frequency fluctuations. Also, the mode mismatch between the fiber and the cavity mode led to a low cavity output efficiency of approximately 4 %.

We circumvent these problems by directly patterning PnC structures on both the top and bottom mirrors. The top and bottom mirror substrates are respectively fused silica and borosilicate glass, with a high-reflection coating sputtered on one side and an anti-reflection layer coating the other side. We use a dicing saw for glass machining to dice a regular array of lines into the mirror substrates. The blade is continuously cooled by a pressurized water jet during the dicing process. The maximum cut depth allowed for our blade is 2.5 mm and we constrain the designed PnC accordingly. We cut the flat bottom mirror only from one side (its thickness is only 1 mm), and the top mirror is diced symmetrically with parallel cuts from both sides, since it is 4-mm thick. The relatively deep cuts in the top mirror need to be patterned over multiple passes, with gradually increasing depths. After dicing one mirror side, the piece is flipped and the other side is diced after aligning to the first cuts, visible through the glass substrate. Lines are arranged in a square lattice for simplicity (see Fig. 2(d,f)), although more complex patterns can be machined with the dicing saw. We simulate the band diagrams of the unit cells of both the top and the bottom mirrors in COMSOL Multiphysics with the Structural Mechanics module. The simulation result is shown in Fig. 2 of the main text. We optimized the lattice constant and cut depths in order to maximize the bandgap width, while centering the bandgap around 1 MHz and making sure that the remaining glass thickness is sufficient to maintain a reasonable level of structural stiffness.

Due to the finite size of the mirrors, we expect to observe edge modes within the mechanical bandgap frequency range. The thermal vibrations of these modes penetrate into the PnC structure with exponentially decaying amplitudes. To account for their noise contributions, we simulated the frequency noise spectrum of the MIM assembly, consisting of the bottom and the top mirrors in contact with a silicon spacer chip, shown in Fig. J.1. Displacement noise at the location of the cavity optical mode is estimated using the fluctuation-dissipation theorem and the eigenmode parameters obtained from the COMSOL eigenfrequency solver, then converted to frequency noise. From previous measurements of mirror modes, we assume a uniform quality factor of 10^3 for all the modes. The eigenfrequency solution confirmed the existence of edge modes with frequencies within the mechanical bandgap, but did not predict any significant contribution to the cavity frequency noise: the PnC is sufficiently large to reduce their contribution at the cavity mode position. The bandgap noise is mostly contributed from the off-resonant tail of the thermal mechanical noise from the modes outside the bandgap. We also observe that the noise peaks at the upper edge of the bandgap are sensitive to the relative displacement of the optical mode from the mirror center (see Fig. J.1). This is attributed to the spatial symmetries of these modes.

After patterning the PnC structures on the mirrors, we assembled a cavity with a spacer chip in place of a membrane, and observed that the TE_{00} linewidth with the diced mirrors is identical to that of the original cavity. This indicates that our fabrication process does not

cause measurable excess roughness or damage to the mirror surfaces. On the other hand, we noticed that when the assembly was clamped too tightly, excess cavity loss occurred due to significant deformation of the PnC mirrors, with a reduced stiffness. We mitigate this detrimental effect in the experiment by gently clamping the MIM cavity, with a spring tension sufficient to guarantee the structural stability of the assembly. We also ensure that the cavity mode is well-centered on the bottom mirror, to reduce the aforementioned upper band-edge modes thermal noise contribution.

To characterize the thermal mechanical noise of the cavity mirrors, we assemble a MIM cavity with the membrane chip replaced by a spacer chip. Similar to the optomechanical g_0 calibration [2], a phase-modulation tone is used as a reference for calibrating the noise in cavity frequency unit. The displacement noise is converted from the cavity frequency noise using the cavity pulling factor $\delta f = \delta x \cdot f_{\mathrm{cav}}/L$, where δf is the cavity frequency noise, δx is the mirror displacement noise, f_{cav} is the cavity mode frequency, and L is the cavity length. For the MIM experiment discussed in the main text, we did not observe any mirror modes within the mechanical bandgap of the membrane chip. We can distinguish membrane modes from mirror modes by exploiting the fact that the coupling rates of membrane modes vary between different cavity resonances, while this is not the case for mirror modes.

For the assembly of the MIM system, apart from the gentle clamping of the phononic crystal mirrors to avoid mirror deformation, and the centering of the optical mode on the bottom mirror (with an optical microscope for mode imaging) to reduce cavity noise on the upper band edge, special attention needs to be paid to the cleaning of the backside of the membrane chip. We clean the backside of the membrane chip using cleanroom Q tip with aceton, to ensure that the membrane is as parallel as possible to the bottom mirror so as to reduce tilting-induced membrane scattering when they are in hard contact. Given the physical dimensions of the MIM, including the membrane chip thickness (distance from the membrane to the bottom mirror) 525 µm, the distance from the membrane to the top mirror 110 µm, the cavity length 635 µm, the membrane thickness 20 nm, we found through simulation that the cavity linewidth modulation from the field redistribution effect of the membrane is at maximum 10%. This is comparably much smaller than the observed cavity linewidth modulation shown in Fig.2(b). We suspect that this could be due to the membrane-surface roughness-induced scattering, and the residual tilting of the membrane that can not be compensated by the backside cleaning.

Section K: TiSa laser phase noise characterization

We upper-bound the phase noise of the TiSa laser (Sirah Lasertechnik) used in the experiment by beating it with another TiSa laser (M Squared). The frequency difference of the two lasers is passively stable around 9 MHz, and the beating signal is recorded digitally with a sampling rate of 56 MHz. The signal is then demodulated at 9 MHz to retrieve the I/Q data, from which the phase noise is retrieved. The noise densities of both the laser phase and frequency are computed and shown in Fig. K.1. At 1 MHz where the experiment is conducted, the measured noise density is primarily limited by the detector noise and laser shot noise. Therefore, we upper-bound the laser frequency noise at $\bar{S}_{\nu\nu}(1\,\mathrm{MHz}) < 3 \times 10^{-2}\,\mathrm{Hz}^2/\mathrm{Hz}$.

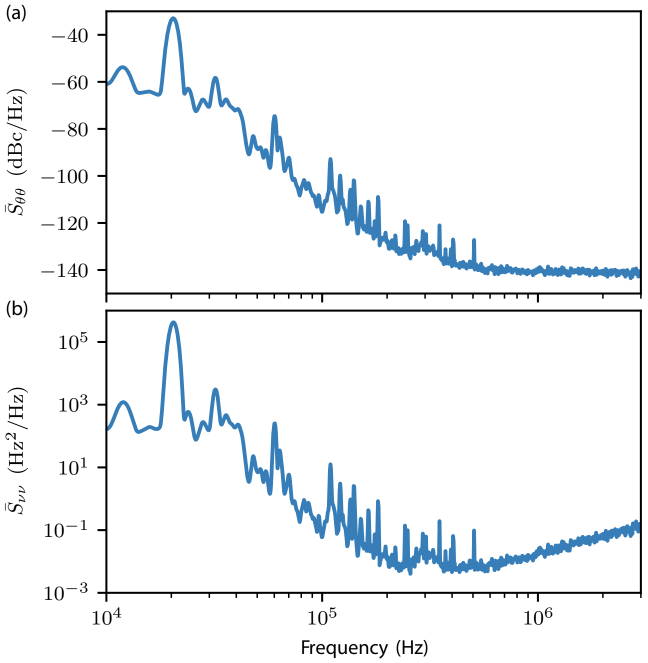

FIG. K.1. Measured noise density of the laser relative **(a)** phase and **(b)** frequency fluctuations of the two TiSa lasers.

[1] M. Aspelmeyer, T. J. Kippenberg, and F. Marquardt, Reviews of Modern Physics **86**, 1391 (2014).
[2] M. L. Gorodetksy, A. Schliesser, G. Anetsberger, S. Deleglise, and T. J. Kippenberg, Optics Express **18**, 23236 (2010).
[3] S. Weis, R. Rivière, S. Deléglise, E. Gavartin, O. Arcizet, A. Schliesser, and T. J. Kippenberg, Science **330**, 1520 (2010).
[4] W. P. Bowen and G. J. Milburn, *Quantum Optomechanics* (CRC Press, Boca Raton, 2015).

[5] L. Magrini, P. Rosenzweig, C. Bach, A. Deutschmann-Olek, S. G. Hofer, S. Hong, N. Kiesel, A. Kugi, and M. Aspelmeyer, Nature **595**, 373 (2021).
[6] J. Cripe, N. Aggarwal, R. Lanza, A. Libson, R. Singh, P. Heu, D. Follman, G. D. Cole, N. Mavalvala, and T. Corbitt, Nature **568**, 364 (2019).
[7] K. Jacobs and P. L. Knight, Physical Review A **57**, 2301 (1998).
[8] U. Andersen, G. Leuchs, and C. Silberhorn, Laser & Photonics Reviews **4**, 337 (2010).
[9] J. Zhang and K. Mølmer, Physical Review A **96**, 062131 (2017).
[10] F. Massel, S. U. Cho, J.-M. Pirkkalainen, P. J. Hakonen, T. T. Heikkilä, and M. A. Sillanpää, Nature Communications **3**, 987 (2012).
[11] J. Guo, R. Norte, and S. Gröblacher, Physical Review Letters **123**, 223602 (2019).
[12] S. A. Saarinen, N. Kralj, E. C. Langman, Y. Tsaturyan, and A. Schliesser, Optica **10**, 364 (2023).
[13] S. A. Fedorov, N. J. Engelsen, A. H. Ghadimi, M. J. Bereyhi, R. Schilling, D. J. Wilson, and T. J. Kippenberg, Physical Review B **99**, 054107 (2019).
[14] A. Beccari, D. A. Visani, S. A. Fedorov, M. J. Bereyhi, V. Boureau, N. J. Engelsen, and T. J. Kippenberg, Nature Physics **18**, 436 (2022).
[15] M. J. Bereyhi, A. Arabmoheghi, A. Beccari, S. A. Fedorov, G. Huang, T. J. Kippenberg, and N. J. Engelsen, Physical Review X **12**, 021036 (2022).
[16] P.-L. Yu, TP. Purdy, and CA. Regal, Phys. Rev. Lett. **108**, 083603 (2012).
[17] Y. Tsaturyan, A. Barg, E. S. Polzik, and A. Schliesser, Nature Nanotechnology **12**, 776 (2017).
[18] D. Høj, U. B. Hoff, and U. L. Andersen, Ultra-coherent nanomechanical resonators based on density phononic crystal engineering (2022), arxiv:2207.06703 [cond-mat].
[19] M. Bao, H. Yang, H. Yin, and Y. Sun, Journal of Micromechanics and Microengineering **12**, 341 (2002).
[20] K. An, B. A. Sones, C. Fang-Yen, R. R. Dasari, and M. S. Feld, Optics Letters **22**, 1433 (1997).
[21] S. A. Fedorov, A. Beccari, A. Arabmoheghi, D. J. Wilson, N. J. Engelsen, and T. J. Kippenberg, Optica **7**, 1609 (2020).
